# Fibroblast dynamics in colorectal cancer: stability, plasticity, and novel markers

**DOI:** 10.1038/s41388-026-03809-6

**Published:** 2026-04-28

**Authors:** Richard Demmler, Charles G. Anchang, Yongsong Yong, Andreas Ramming, Simon Rauber, Vera S. Schellerer, Benjamin Schmid, Arndt Hartmann, Susanne Merkel, Katharina Imkeller, Elisabeth Naschberger, Michael Stürzl

**Affiliations:** 1 Division of Molecular and Experimental Surgery, Uniklinikum Erlangen, Friedrich-Alexander-Universität Erlangen-Nürnberg (FAU), Erlangen, Germany; 2 Department of Medicine 3 - Rheumatology and Immunology, Friedrich-Alexander-Universität Erlangen-Nürnberg, Uniklinikum Erlangen, Erlangen, Germany; 3 Deutsches Zentrum für Immuntherapie (DZI), Friedrich-Alexander-Universität Erlangen-Nürnberg, Uniklinikum Erlangen, Erlangen, Germany; 4 Institute of Neurology (Edinger Institute), Goethe University, University Hospital Frankfurt, Frankfurt/Main, Germany; 5 FAU Competence Centre Optical Imaging Centre Erlangen, Friedrich-Alexander-Universität Erlangen-Nürnberg (FAU), Erlangen, Germany; 6 Institute of Pathology, Uniklinikum Erlangen, Friedrich-Alexander-Universität Erlangen-Nürnberg (FAU), Erlangen, Germany; 7 CCC Erlangen-EMN: Comprehensive Cancer Center Erlangen-EMN (CCC ER-EMN), Erlangen, Germany; 8 CCC WERA: Comprehensive Cancer Center Alliance WERA (CCC WERA), Erlangen, Germany; 9 BZKF: Bavarian Cancer Research Center (BZKF), Erlangen, Germany; 10 Department of Surgery, Uniklinikum Erlangen, Friedrich-Alexander-Universität Erlangen-Nürnberg (FAU), Erlangen, Germany; 11 University Cancer Center (UCT), Frankfurt/Main, Germany; 12 Frankfurt Cancer Institute (FCI), Frankfurt/Main, Germany; 13 Present Address: Department of Pediatric Surgery, University of Greifswald, Greifswald, Germany

**Keywords:** Cancer microenvironment, Biomarkers, Sequencing

## Abstract

Colorectal cancer (CRC) is one of the most commonly diagnosed and globally spread malignant diseases. Cancer-associated fibroblasts (CAFs) are key architects of the tumor microenvironment, yet their origin, stability, and interconvertibility remain poorly understood. Using transcriptomic profiling of fibroblasts from colorectal cancer (CRC) patients, we identify highly expressed (HEX) markers that define fibroblast subpopulations and uncover mechanisms governing their plasticity. We find that ADH1B marks normal colon-associated fibroblasts (NAFs), which consist of PI16-NAFs and ADAMDEC1-NAFs. ITGA3 delineates the total CAF population, which comprises myofibroblastic CAFs (myCAFs), whose characterizing markers were associated with poor prognosis and proteolytic inflammatory CAFs (piCAFs), characterized by markers not associated with prognosis. An AGT/TGM2-expressing fibroblast subset is present in both healthy and tumor tissues, suggesting alternative trajectories to the classical NAF-to-CAF transition model. While PI16-NAFs, AGT/TGM2-fibroblasts, and myCAFs maintain stable identities in long-term culture, the ADAMDEC1-NAF and piCAF phenotypes are lost in vitro. ITGA3-CAFs demonstrate dynamic plasticity, with TGF-β stably inducing myCAF formation and TNF-α or inhibition of DNA methylation promoting transient piCAF emergence. These findings redefine fibroblast heterogeneity in CRC and reveal a coexisting stable and plastic fibroblast network that may be amenable to modulation and provides a framework for future functional and translational studies.

**Figure:**
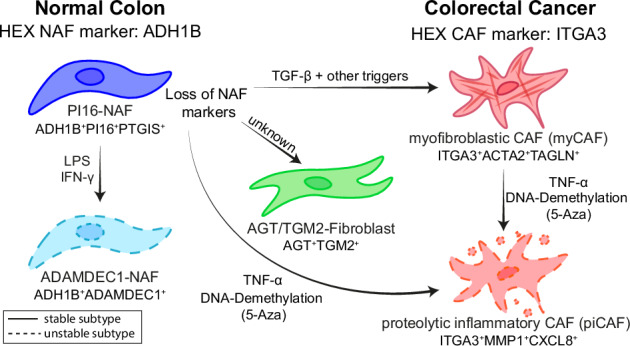
We identified highly expressed markers (HEX markers) to distinguish CAFs, NAFs and corresponding subpopulations in CRC. ADH1B characterized NAFs, which consisted of stable (solid outline) PI16-NAFs and unstable (dashed outline) ADAMDEC1-NAFs. ITGA3 identified CAFs consisting of stable myCAFs associated with poor prognosis and unstable piCAFs not associated with prognosis. AGT/TGM2 fibroblasts did not express ADH1B or ITGA3, were stable in culture and could be detected in both healthy colon and CRC. Treatment of PI16-NAFs with LPS or IFN-γ induced ADAMDEC1-NAFs, TGF-β the formation of myCAFs, while treatment with TNF-α led to the formation of piCAFs. Reduced DNA methylation converted myCAFs and PI16-NAFs into piCAFs.

We identified highly expressed markers (HEX markers) to distinguish CAFs, NAFs and corresponding subpopulations in CRC. ADH1B characterized NAFs, which consisted of stable (solid outline) PI16-NAFs and unstable (dashed outline) ADAMDEC1-NAFs. ITGA3 identified CAFs consisting of stable myCAFs associated with poor prognosis and unstable piCAFs not associated with prognosis. AGT/TGM2 fibroblasts did not express ADH1B or ITGA3, were stable in culture and could be detected in both healthy colon and CRC. Treatment of PI16-NAFs with LPS or IFN-γ induced ADAMDEC1-NAFs, TGF-β the formation of myCAFs, while treatment with TNF-α led to the formation of piCAFs. Reduced DNA methylation converted myCAFs and PI16-NAFs into piCAFs.

## Introduction

Colorectal cancer (CRC) remains a major global health burden, ranking as the third most common cancer worldwide and a leading cause of cancer-related mortality [1]. Fibroblasts are among the most abundant cell types in the tumor stroma and play a key role in CRC progression by modulating the tumor microenvironment (TME) through extracellular matrix remodeling and cytokine-driven intercellular communication, also influencing therapeutic responses [2–6].

However, the specificity of proposed fibroblast and subpopulation markers remains controversial. These discrepancies largely stem from the fact that CAF markers are often identified solely through transcriptomic analyses of tumor tissues without inclusion of normal fibroblasts (NAFs) as a reference, and without assessing whether computationally defined clusters represent real and at least transiently stable subpopulations. Additionally, while many reported markers demonstrated statistical significance, their expression levels were often too low to allow reliable single-cell detection in tissues with immunostainings. Given these considerations, the identification of specific markers and a deeper understanding of fibroblast heterogeneity, stability, and interconversion within the TME have emerged as critical goals [4]. Using paired CAFs and NAFs isolated from individual patients, we identified highly expressed markers (HEX markers) enabling sensitive detection of CAFs and NAFs at the single-cell level in human CRC tissues and cultured cells. This approach uncovered five subpopulations with different stability and prognostic associations. Moreover, we provide evidence for epigenetic and cytokine-mediated regulation of subtype development. These findings open new perspectives for potential therapeutic targeting of fibroblast subpopulations.

## Methods

### Ethics approval and consent to participate

Approval for all patient-related studies was obtained from the local ethics committee (institutional review board) of the FAU Erlangen-Nürnberg (approval number: TuMiC study, #159 15 B). All participants were informed personally and provided written consent. Patient data were pseudonymized, and all procedures complied with the Declaration of Helsinki.

### Isolation and cell culture of human NAFs and CAFs

Human NAFs and CAFs were isolated from CRC patients following established procedures [7–10]. The inclusion criteria were CRC (stage I–IV) without prior treatment. Patients with inflammatory bowel disease or known familial predisposition were excluded. Detailed patient characteristics and specific experimental allocations are listed in Supplementary Tables S1, S2. In brief, CAFs were obtained from the non-necrotic tumor center. NAFs were isolated from healthy tissue at least 10 cm from the tumor margin. Approximately 5–7 days after initial seeding, endothelial cells were removed by magnetic cell separation using CD31 beads. The resulting negative fraction (=fibroblast fraction) was cultured in DMEM supplemented with 10% FCS (Sigma-Aldrich, St. Louis, Missouri, USA; #S0615), 1% L-Glutamine (Thermo Fisher, Waltham, Massachusetts, USA; #25030-024) and 1% Penicillin/Streptomycin (Gibco, Waltham, Massachusetts, USA; #15140-122) at 37 °C with 8.5% CO_2_ and 95% humidity until reaching passage 7 (splitting 1:4 equaled one passage). Medium was changed twice weekly, and cells were used at 80–90% confluence. Cells were routinely tested for mycoplasma contamination and characterized by staining for CD31, CK20 and CD45 (all negative) and CD105/vimentin (positive) expression [7].

### Statistical analyses

Two-tailed unpaired *t*-tests were performed to validate the expression of DEGs in CAFs and NAFs; paired *t*-tests assessed collagen remodeling upon 5-Aza or cytokine treatment. RT-qPCR data were analyzed using the ddCT method and ratio-paired *t*-tests. A Gaussian distribution was assumed in all cases. Spearman’s correlation was used for gene association analyses. GraphPad Prism v10.20 (GraphPad Software, Inc.) was used, with *p* < 0.05 considered significant. RNA-seq results were evaluated using adjusted *p* values. All graphs show mean ± standard deviation (SD).

All other methods can be found in the supplemental information due to word count constraints.

## Results

### Fibroblasts isolated from peritumoral normal tissue and human colorectal cancer tissue stably maintain phenotypic differences in vitro and can be distinguished by *ADH1B* and *ITGA3* expression

**Fig. 1:**
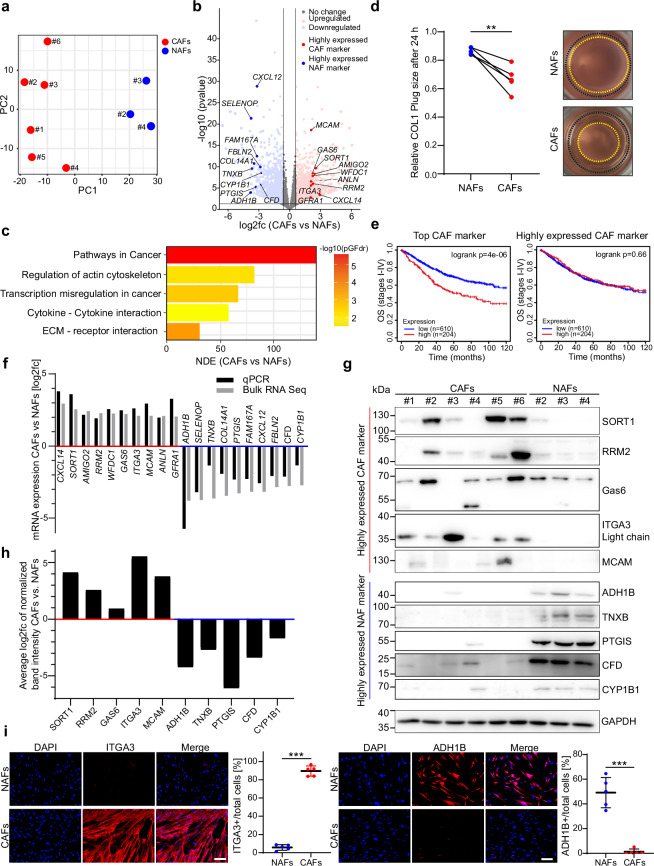
Isolated NAFs and CAFs exhibit stable phenotypic differences in vitro. **a** Principal component analysis of the bulk RNA-seq data of cultured CAFs (*n* = 6; #1–6) and NAFs (*n* = 3; #2–4). **b** Volcano plot depicting the significantly deregulated genes (*p* < 0.05, fold change >1.5) between CAFs and NAFs. Highly expressed (HEX) NAF and CAF differentiation markers are indicated. **c** Bar plot displaying the negative log10 false discovery rate (FDR) and number of differentially expressed genes (NDE) of the most significantly upregulated KEGG2020 terms enriched in isolated and cultured CAFs according to conducted bulk RNA-seq analysis. **d** Assessment of paired CAFs and NAFs to remodel a rat tail collagen 1 plug depicted by plug shrinkage after 24 h (*n* = 5; #3, #7, #8, #9, #10; paired *t*-test, **P < 0.01). **e** High expression of the top CAF differentiation markers is associated with poorer overall survival (left) in colorectal cancer, whereas the expression of the HEX CAF markers is not prognostic for OS (right). High expression was defined using the upper quartile of the total cohort. **f** Validation of HEX NAF and CAF markers by independent RT-qPCR. Comparison of the log2fc in CAFs (*n* = 6; #1–6) vs NAFs (*n* = 3; #2–4) measured by RT-qPCR (black) and bulk RNA-seq (gray) is displayed. **g** Validation of HEX NAF and CAF markers on protein level by Western Blot. **h** Quantitative evaluation of Western blot results in (**g**). Signal intensity was normalized to the respective GADPH bands in CAFs (*n* = 6; #1–6) compared to NAFs (*n* = 3; #2–4) with the average log2fc displayed. **i** Immunocytochemical detection of ITGA3 (left) and ADH1B (right) representing HEX-markers for NAFs and CAFs, respectively. For quantitative analyses total cell count and number of positive cells were determined using ImageJ and averaged among three random areas per sample in paired NAFs and CAFs (*n* = 5, #2, #3, #8, #11, #12). Scalebar represents 150 μm (paired two-tailed *t*-test, ***P < 0.001). #, patient numbers as detailed in Supplementary Tables S1, S2.

CAFs (*n* = 6) and corresponding NAFs (*n* = 3) were isolated from human treatment-naive CRC patients and briefly expanded (<2 passages) to provide sufficient material for analyses and validation. Subsequently, the transcriptomes of CAFs and NAFs were compared via bulk RNA-sequencing (Supplementary Tables S1, 2). Principal component analysis revealed clear differences between CAFs and NAFs (Fig. 1a). A total of 1528 genes were upregulated and 1418 genes were downregulated in CAFs (Fig. 1b), resulting in changes in disease-related pathways such as “*Pathways in cancer”*, *“Actin cytoskeleton regulation”*, *“Misregulated transcription”*, *“Cytokine‒cytokine interaction”* and *“ECM‒receptor interaction*” (Fig. 1c). Notably, CAFs retained robust functional ability for increased collagen 1 remodeling in long-term cell culture (Fig. 1d), which is a well-established hallmark of CAFs [4, 11].

**Fig. 2:**
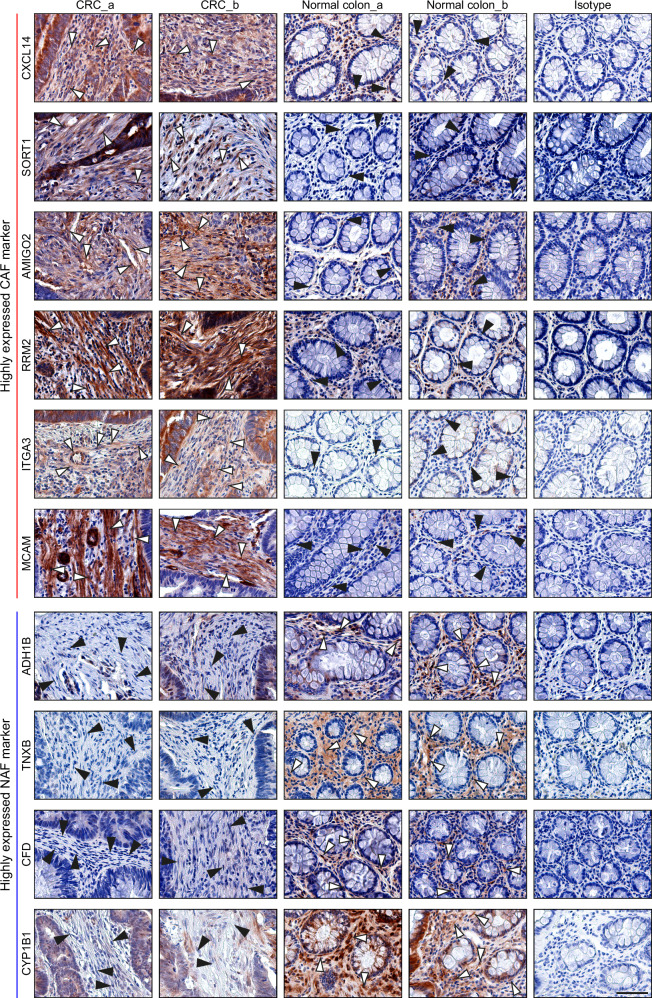
Distinct NAF and CAF signatures are validated by staining of normal colon and CRC tissues. Immunohistochemical detection of the highly expressed CAF (CXCL14, SORT1, AMIGO2, RRM2, ITGA3 and MCAM) and NAF (ADH1B, TNXB, CFD, CYP1B1) markers on FFPE-tissue sections of tumor stroma areas and healthy colon. Two representative pictures of paired CRC and normal colon tissues from the same patients (patients a and b) and an isotype control are depicted. White arrowheads indicate positive fibroblasts, black arrowheads indicate fibroblasts negative for the respective marker. Scalebar represents 100 μm.

The gene signatures of the 10 most differentially expressed protein-coding genes in CAFs (TOP CAF marker, Supplementary Table 3) ranked by log2fc were associated with a poorer overall survival (OS), especially in late-stage patients and significantly associated with worse relapse-free survival (RFS), independent on tumor stage (Fig. 1e, left and Supplementary Fig. 1a). However, these genes generally exhibited low RNA expression (see Top marker, Supplementary Table 3) and were therefore not suitable as markers for the reliable detection of CAFs at the single-cell level in tissue sections. To identify more applicable markers, we set a threshold of 5000 normalized mean reads as the minimal expression level for genes and selected the 10 most up- and downregulated genes, establishing a panel of highly expressed (HEX) CAF and NAF markers. CAF HEX markers according to these criteria included *C-X-C motif chemokine ligand 14* (*CXCL14*), *sortilin 1* (*SORT1*), *adhesion molecule with Ig like domain 2* (*AMIGO2*), *ribonucleotide reductase regulatory subunit M2* (*RRM2*), *WAP four-disulfide core domain 1* (*WFDC1*), *growth arrest specific 6* (*GAS6*), *integrin subunit alpha 3* (*ITGA3*), *melanoma cell adhesion molecule* (*MCAM*), *anillin, actin binding protein* (*ANLN*) and *GDNF family receptor alpha 1* (*GFRA1*), while the selected NAF markers included *alcohol dehydrogenase 1B* (*ADH1B*), *selenoprotein P* (*SELENOP*), *tenascin XB* (*TNXB*), *collagen type XIV alpha 1 chain* (*COL14A1*), *prostaglandin I2 synthase* (*PTGIS*), *family with sequence similarity 167 member A* (*FAM167A*), *C-X-C motif chemokine ligand 12* (*CXCL12*), *fibulin 2* (*FBLN2*), *complement factor D* (*CFD*) and *cytochrome P450 family 1 subfamily B member 1* (*CYP1B1*) (Fig. 1b and Supplementary Table S3). Unlike the Top CAF marker, the HEX CAF marker panel was no longer able to predict overall survival (Fig. 1e, right), but was prognostic for relapse-free survival (RFS) in late-stage patients (Supplementary Fig. 1b). Of note, the differential expression between NAFs and CAFs was confirmed at the RNA level by independent RT-qPCR for all the target genes (Fig. 1f) and, most importantly, for 10 genes at the protein level (Fig. 1g, quantitative analyses Fig. 1h). In addition, ITGA3 as a promising HEX CAF and ADH1B as the corresponding NAF marker were validated at the single-cell level in cultured fibroblasts, confirming their differential expression in vitro (Fig. 1i). The six HEX CAF markers (CXCL14, SORT1, AMIGO2, RRM2, ITGA3, and MCAM) and four HEX NAF markers (ADH1B, TNXB, CFD, and CYP1B1) were validated in normal colon and CRC tissues (Fig. 2). Further analyses indicated that the HEX CAF markers CXCL14, SORT1, AMIGO2, RRM2 and MCAM were not fully appropriate, because they were either present in a small fraction of cells in the normal colon in vivo or not expressed in CAF cultures from all patients in vitro (data not shown). In contrast, ITGA3 consistently discriminated CAFs from NAFs, both in vitro (Fig. 1i) and in vivo (Fig. 2), and accordingly established a new HEX marker for CAFs.

Similarly, the most reliable marker among the remaining four HEX NAF markers was determined. Analysis of an external scRNA-seq dataset of CRC tissues indicated that CYP1B1 may not be entirely specific, as it was sporadically expressed in CAFs (in vivo cohort, see below). Moreover, TNXB and CFD are secreted proteins, which may yield less cell-associated detection. Conversely, ADH1B is a cell-retained cytoplasmic protein and was exclusively detected in NAF cultures (Fig. 1i). Based on these results, ADH1B and ITGA3 were found to be the most reliable HEX markers that allow differentiation of NAFs and CAFs at the single cell level in culture (Fig. 1i) and in vivo (Fig. 2).

### Identification of fibroblast subpopulations in CRC

**Fig. 3:**
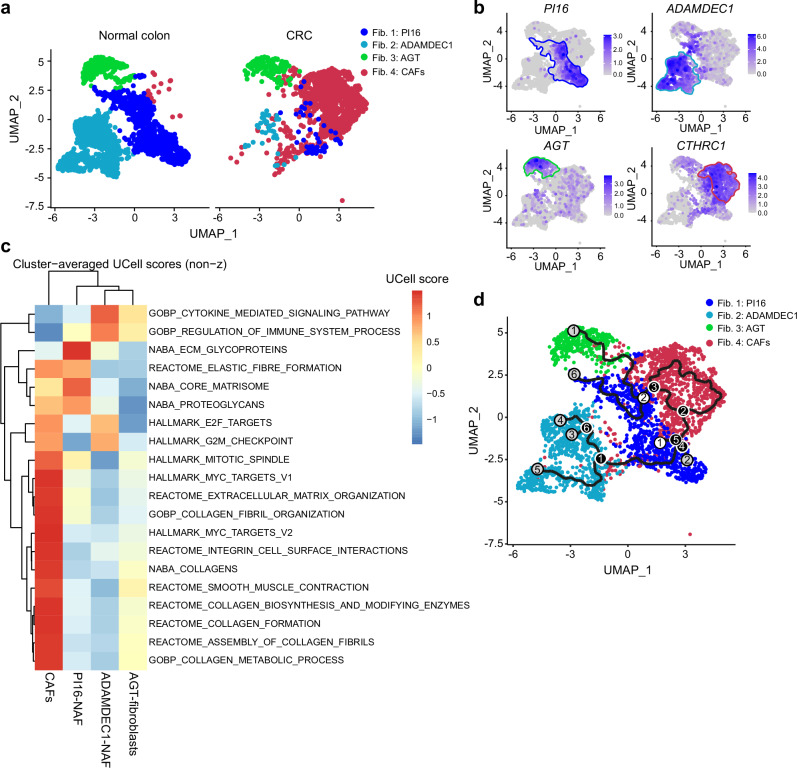
Identification of fibroblast subtypes in CRC. **a** Description of fibroblast subpopulations extracted from a CRC vs. normal colon scRNA-seq dataset [12]. PI16 and ADAMDEC1 define NAF subpopulations, whereas the AGT-subpopulation is present in both, CAFs and NAFs. CAFs represent the major fibroblast population in CRC tissues. **b** PI16, ADAMDEC1, AGT and CTHRC1 are markers of the respective fibroblast subpopulations. **c** Cluster-averaged UCell scores displaying association of pathways with respective fibroblast subtypes. **d** Pseudotime analysis displaying trajectories originating from PI16-NAFs. White-filled circles display the trajectory root, black-filled circles branching points and gray-filled circles terminal endpoints.

To identify potential subpopulations of ADH1B-NAFs and ITGA3-CAFs, a publicly available single-cell transcriptomic dataset of 23 CRC and 10 matching normal colon tissues (in vivo cohort) was re-analyzed [12]. After filtering out low-quality cells, 57,927 single cells were subjected to UMAP clustering to distinguish stromal cells from epithelial, immune and myeloid cells (Supplementary Fig. 2a). Fibroblasts were distinguished from other stromal cells using known markers including *lumican* (*LUM*), *decorin* (*DCN)*, *fibulin 1* (*FBLN1)* and *collagen type I alpha 1 chain* (*COL1A1*) (Supplementary Fig. 2b, c) [12–14]. All the fibroblasts co-expressed the characteristic pan-fibroblast markers *COL1A1* and *COL1A2* (Supplementary Fig. 2d). By grouping similar clusters, we identified four distinct fibroblast subpopulations (Fib. 1–4), while a fifth cluster expressing *serpin family B member 2* (*SERPINB2*), present in only one patient, was excluded (Supplementary Fig. 2e–f). The remaining clusters were annotated using their most characteristic genes, while taking recently reported markers into account (Fig. 3a, b, and Supplementary Fig. 2g) [14]. Fib. 1 and Fib. 2, marked by high *peptidase inhibitor 16* (*PI16*) and *ADAM like decysin 1* (*ADAMDEC1*) expression, respectively, were mostly restricted to normal colon tissues (Fig. 3a, left). Fib. 3, defined by *angiotensinogen* (*AGT*) expression was detected in both normal colon and CRC tissues (Fig. 3a). Fib. 4, expressing *CTHRC1*, was almost exclusively found in CRC tissues (Fig. 3a, right) and was thus designated the CAF population.

Cluster-averaged UCell scores revealed a clear separation between subtypes, indicating distinct functional specializations (Fig. 3c). PI16-NAFs showed selective enrichment for ECM glycoprotein, proteoglycan, and core matrisome pathways and demonstrated the capacity to differentiate into all other subtypes (Fig. 3d), consistent with a progenitor-like fibroblast state. ADAMDEC1-NAFs, upregulated cell-cycle related pathways, cytokine-mediated signaling and immune regulatory pathways, identifying them as a proliferative and immune-modulatory NAF population. AGT-fibroblasts displayed upregulation of extracellular matrix and collagen programs relative to the other NAFs, pointing towards regulation of the ECM homeostasis in the healthy colon. These ECM signatures were further amplified in the CAF cluster, which showed the strongest enrichment for matrix-remodeling and collagen pathways, while downregulating immune interaction processes.

**Fig. 4:**
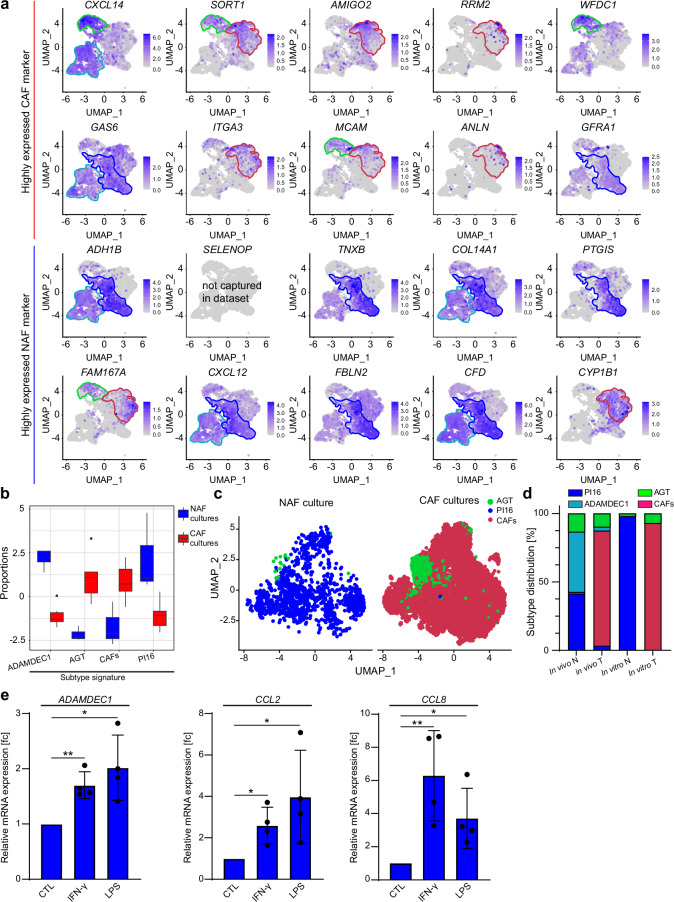
Distinct subpopulations of human NAFs and CAFs isolated from CRC patients are preserved in vitro. **a** UMAP plots displaying the expression of the highly expressed CAF and NAF differentiation markers in the four identified fibroblast subpopulations. The subpopulations with the highest relative expression are circled. NAF differentiation markers are mainly associated with PI16-NAFs (dark blue) and ADAMDEC1-NAFs (light blue). CAF differentiation markers are more abundant in the CAF cluster (red) and the AGT-subpopulation (green). **b** Distribution of the four identified subpopulations in cultured NAFs and CAFs based on decomposition of the bulk RNA-seq data from cultivated CAFs and NAFs. ADAMDEC1- and PI16-NAF gene signatures are enriched in NAF cultures, whereas the AGT-fibroblast and the CAF signature are more abundant in CAF cultures. **c** Integration-based label transfer of the identified fibroblast subpopulations onto a scRNA-seq dataset of cultured fibroblasts isolated from CRC and normal colon tissues identifies stable fibroblast phenotypes in cultured CAFs and NAFs (CAFs: 14,967 cells from *n* = 3 samples: #2, #3, #6; NAF: 1581 cells from *n* = 1 sample: #3). PI16-NAFs are preserved in NAF cultures, CAF cultures are made up by CAFs and AGT-fibroblasts. **d** Comparison of the distribution of the four fibroblast subpopulations in vivo (normal colon and CRC) and in vitro (cultured NAFs and CAFs). **e** Induction of *ADAMDEC1*, *CCL2* and *CCL8* by stimulation with LPS (1 µg/ml) and IFN-γ (5 U/ml) in NAFs (*n* = 4: #3, #7, #9, #13). Gene expression was determined by RT-qPCR and normalized to untreated cells (Ratio-paired two-tailed *t*-test, *P < 0.05, **P < 0.01). #, patient numbers as detailed in Supplementary Tables S1, S2.

In the next step, the expression of the HEX and Top markers was assigned to the four fibroblast subpopulations (Fig. 4a and Supplementary Fig. 3a). Notably, the majority of the HEX CAFs markers including *AMIGO2*, *RRM2*, *ITGA3* and *ANLN* were exclusively associated with CAFs in the in vivo cohort (Fig. 4a, Table 1). In contrast, the HEX markers for NAFs were predominantly expressed in the two NAF subpopulations (Fig. 4a, Table 1). *ADH1B*, *CXCL12* and *CFD* were associated with both, PI16-NAFs and ADAMDEC1-NAFs, whereas *TNXB*, *PTGIS* and *FBLN2* specifically characterized PI16-NAFs (Fig. 4a, Table 1). No marker was identified that was specific for ADAMDEC1-NAFs (Fig. 4a, Table 1). The HEX CAF marker *WFDC1* was found to be restricted to AGT-fibroblasts (Fig. 4a, Table 1).

**Table 1:** Association between the highly expressed markers of cultivated NAF and CAF and different fibroblast subtypes.

	Gene	Fib. 1 PI16	Fib. 2 ADAMDEC1	Fib. 3 AGT	Fib. 4 CAF
Highly expressed CAF marker	*CXCL14*		1.44	1.59	
	*SORT1*			1.19	2.06
	*AMIGO2*				2.66
	*RRM2*				3.91
	*WFDC1*			2.63	
	*GAS6*	0.89	0.16^*^		
	*ITGA3*				2.13
	*MCAM*			2.25	0.90
	*ANLN*				4.12
	*GFRA1*	2.50			
Highly expressed NAF marker	*ADH1B*	2.09	0.63		
	*SELENOP*	N/A	N/A	N/A	N/A
	*TNXB*	2.99			
	*COL14A1*	1.73	0.15		
	*PTGIS*	3.28			
	*FAM167A*			0.58^*^	3.09
	*CXCL12*	1.39	0.52		
	*FBLN2*	1.45			
	*CFD*	1.36	1.00		
	*CYP1B1*				4.11

Log2fc for each significantly deregulated gene is displayed for the respective subtype identified from Lee et al. [12] (in vivo cohort).

*N/A* data not available.

^*^p-value <0.01 but adjusted *p*-value >0.01.

Moreover, these analyses confirmed that *ADH1B* and *ITGA3* are novel HEX markers suitable for the identification of NAFs and CAFs at the single-cell level in tissues and isolated cells, respectively (Supplementary Fig. 3b, c). Their specific expression in NAFs and CAFs could be further validated in another scRNA-seq dataset of colorectal cancer (Pelka et al., GSE178341 [15]) (Supplementary Fig. 4a–d). Importantly, increased *ITGA3* levels correlated with poorer RFS regardless of tumor stage (Supplementary Fig. 4e). As epithelial cell–derived ITGA3 likely contributes to this association, ITGA3 may represent a general prognostic factor in CRC.

### Distinct subpopulations of human NAFs and CAFs isolated from CRC patients are preserved in vitro

To determine the long-term stability of the different subpopulations defined above, we estimated their distribution in NAF and CAF cultures. To achieve this goal, the subpopulation signatures were matched with the bulk RNA-seq dataset of cultured NAFs and CAFs. This analysis confirmed that PI16- and ADAMDEC1-fibroblast signature genes were enriched in the NAF cultures, whereas characteristic CAF and AGT-fibroblast genes were more prominently expressed in the CAF cultures (Fig. 4b). To compare the number of different subpopulations in culture quantitatively, a subgroup of the same fibroblast cultures used for bulk RNA-seq were subjected to independent scRNA-seq (CAFs *n* = 3, NAFs *n* = 1) (all passage <2.5) (Supplementary Tables S1, 2). In total, 15,825 cells met the applied quality criteria and were assigned to the signatures of the four subpopulations (PI16, ADAMDEC1, AGT, and CAFs) (Fig. 4c). We identified the PI16-subpopulation in the NAF culture only, whereas the ADAMDEC1-fibroblasts were not detected in any sample (Fig. 4c, d and Supplementary Fig. 5a). In the CAF cultures, 93.1% of the cells preserved the ITGA3-CAF signature; in addition, the AGT-fibroblasts were detected in all the samples and were relatively expanded in the CAF cultures (6.92% of CAFs vs. 1.91% of NAFs) (Fig. 4c, d and Supplementary Fig. 5a).

Comparison of the single-cell transcriptomes from the in vivo cohort and transcriptomes of cultivated cells revealed that the CAF population was robustly increased (in vivo normal: 1.18%, tumor: 84.06%; in vitro NAFs: 0%, CAFs: 93.07%), and the PI16-population was significantly decreased in CRC tissues and cultivated CAFs (in vivo normal: 41.43%, tumor: 3.41%) (Fig. 4d). The AGT-fibroblast population was present in both healthy and CRC tissues in vivo (in vivo normal: 13.30%; tumor: 9.64%) (Fig. 4d).

In contrast, the ADAMDEC1-population was predominant in healthy colon tissue but absent in cultured NAFs (in vivo normal: 44.1%; in vitro NAFs: 0%) (Fig. 4d). Gene expression profiling indicated that ADAMDEC1-NAFs possess higher proliferative potential than PI16-NAFs (Fig. 3c, E2F targets, G2M checkpoint), suggesting that their loss in vitro is unlikely to result from overgrowth by PI16-NAFs but rather from depletion during the isolation process. Consistent with this, ADAMDEC1-fibroblasts were not-detected in the initially isolated, non-passaged NAFs, but were detectable in healthy colon sections (Supplementary Fig. 5b, c).

Most interestingly, ADAMDEC1-NAFs were found to be enriched at the crypt tip (Supplementary Fig. 5b), which is consistent with reports in mouse colitis models showing the presence of a similar ADAMDEC1-fibroblast population in inflammation-associated niches [16]. This spatial localization is consistent with an immunomodulatory phenotype, as ADAMDEC1-NAFs showed upregulation of cytokine-mediated signaling and immune-regulatory pathways and secreted the monocyte-attracting chemokines CCL2 and CCL8 (Supplementary Fig. 2g). Both chemokines are known to be induced by LPS or IFN-γ in monocytes and macrophages [17, 18]. In line with this immune-responsive phenotype, stimulation of NAFs with LPS or IFN-γ induced the key marker genes characteristic of the ADAMDEC1-NAFs (Fig. 4e).

Taken together, these analyses suggested that AGT-fibroblasts, PI16-fibroblasts and CAFs can be stably propagated in culture, whereas ADAMDEC1-fibroblasts are lost during cultivation.

### Increased *TGM2* expression identifies AGT-fibroblasts as a distinct subpopulation present in CAFs and NAFs

**Fig. 5:**
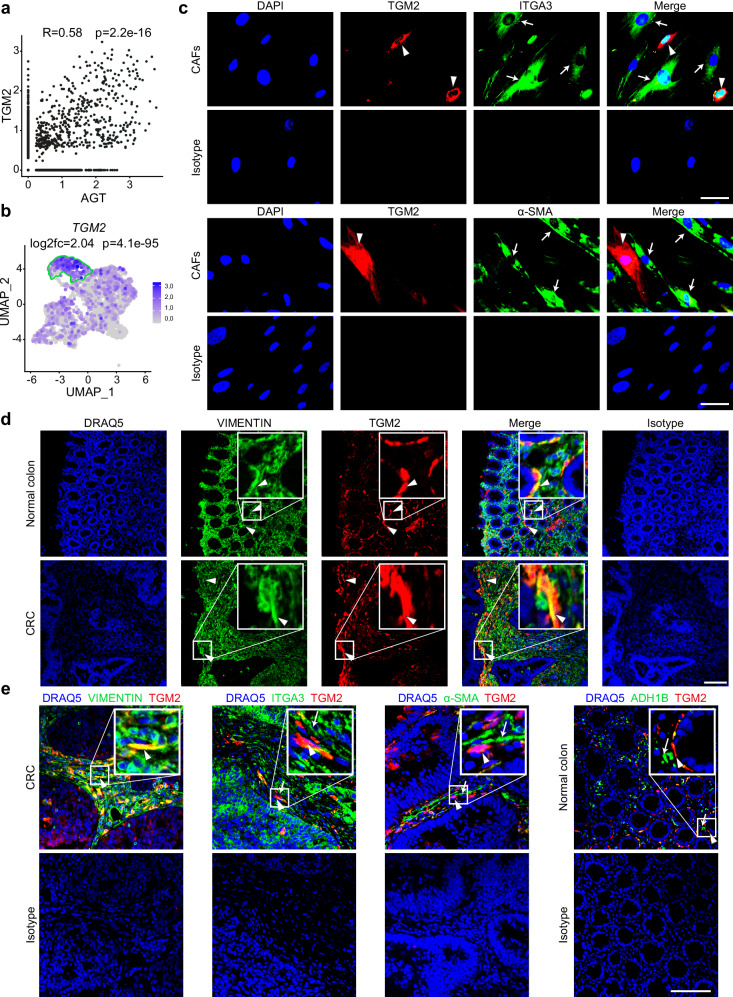
Increased *TGM2* expression identifies AGT-fibroblasts as a distinct subpopulation in CAFs and NAFs. **a** TGM2 is a reliable marker for AGT-fibroblasts as depicted by positive Pearson correlation between TGM2 and AGT in the fibroblasts of the scRNA-seq dataset (23 CRC and 10 matching normal colon tissues) and **b** high expression in these cells. **c** Co-staining of CAFs (CAF#3) with TGM2 and ITGA3 and α-SMA illustrates that the AGT-subpopulation is different from CAFs/myofibroblasts and stably preserved in culture. Arrowheads show TGM2 expressing cells, ITGA3 and α-SMA expressing cells are indicated by arrows. Scalebar represents 50 μm. **d** Stromal TGM2 expression can be found in mesenchymal, vimentin-positive cells both in normal colon and CRC tissues (arrowheads). Scalebar represents 100 μm. **e** Vimentin positive TGM2 expressing fibroblasts (arrowheads) are distinct from ITGA3 and α-SMA expressing CAFs in CRC tissues and ADH1B expressing NAFs in normal colon tissues (arrows). Scalebar represents 250 μm. Nuclei are contrasted with DAPI or DRAQ5 (blue). #, patient numbers as detailed in Supplementary Tables S1, S2.

Previous analyses indicated that AGT-fibroblasts do not express *ADH1B* or *ITGA3* (Fig. 4a and Table 1). However, AGT transcript levels were too low (mean: 43 reads in bulk RNA-seq) to enable reliable detection. Additionally, specific antibodies for the putative AGT-fibroblast marker WFDC1 (Table 1) were not available. To identify an alternative marker, we compared the culture-derived scRNA-seq data with the in vivo cohort. This revealed *transglutaminase 2* (*TGM2*) as a promising candidate, as it was upregulated in AGT-fibroblasts and showed a significant positive correlation with AGT expression (R = 0.58, *p* = 2.2e−16) (Fig. 5a, b).

In accordance with transcriptomic findings, we observed high TGM2 expression in individual cells within CAF cultures (Fig. 5c, arrowheads). Co-staining with the HEX CAF marker ITGA3 revealed that TGM2-positive cells lacked cytoplasmic ITGA3 staining and showed only weak nuclear ITGA3 signal. Conversely, strongly ITGA3-positive cells exhibited cytoplasmic ITGA3 expression but were negative for TGM2 (Fig. 5c, arrows). Further validation with α-smooth muscle actin (α-SMA), a known myofibroblast marker, confirmed that TGM2-expressing cells lacked α-SMA, distinguishing them from the α-SMA-positive CAF population (Fig. 5c).

At the tissue level, TGM2 was expressed in a subpopulation of mesenchymal stromal cells (vimentin-positive) with fibroblast morphology in both normal colon and CRC tissues (Fig. 5d, arrowheads). Moreover, in CRC tissues, vimentin-positive and high-TGM2-expressing fibroblasts expressed neither ITGA3 nor α-SMA (Fig. 5e, arrowheads). Notably, TGM2 expression did not overlap with ADH1B-expressing cells in the healthy colon stroma (Fig. 5e). These findings confirmed that AGT-fibroblasts constitute a distinct subpopulation present in normal colon and CRC tissues, which can be identified by TGM2. Accordingly, we refer to this population as AGT/TGM2-fibroblasts.

High *TGM2* expression was associated with significantly reduced RFS across all stages (log-rank *p* = 0.00052) and remained prognostic in stage I–II patients (log-rank *p* = 0.025), whereas OS demonstrated only a weak association (Supplementary Fig. 6). These findings indicate that elevated TGM2 expression correlates with an increased risk of disease recurrence, particularly in early-stage patients. While TGM2 expression arises from various stromal and epithelial sources, our data support that this fibroblast subtype forms a relevant component of the overall TGM2 signal linked to tumor relapse and progression.

### CAFs divide into myofibroblastic CAFs and proteolytic inflammatory CAFs in human CRC

**Fig. 6:**
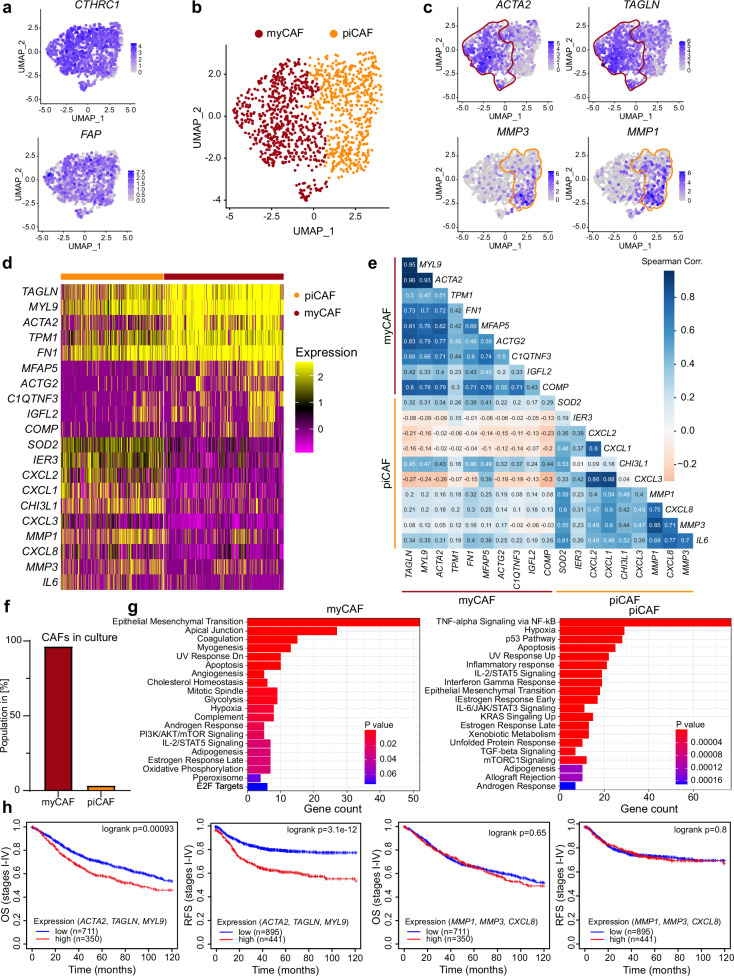
CAFs divide into myofibroblastic CAFs (myCAFs) and proteolytic inflammatory CAFs (piCAFs) in human CRC. **a** *CTHRC1* and *FAP* are evenly expressed by all CAFs. **b** CAFs divide into myofibroblastic CAFs (myCAF) and proteolytic inflammatory CAFs (piCAFs). **c** *ACTA2* and *TAGLN* separate myCAFs from *MMP1* and *MMP3* expressing piCAFs. **d** Heat map of the top-ranking subpopulation specific genes of myCAFs and piCAFs. PiCAFs are characterized by elevated *MMP1*, *MMP3*, *CXCL8* and *IL6* expression. MyCAFs express *ACTA2*, *TAGLN* and *MYL9*. **e** Spearman correlation of mRNA expression of individual genes representative for the piCAF subpopulation with myCAF markers in the COAD tumor and READ tumor cohort. **f** Isolated CAFs in vitro preserve the myCAF phenotype, whereas piCAFs are depleted. **g** Pathway analysis of the most significantly upregulated MSigDB_Hallmark_2020 terms of myCAFs and piCAFs. **h** High expression of myCAF markers (*ACTA2*, *TAGLN*, *MYL9*) is associated with worse prognosis for CRC patients (OS: *p* = 0.00093, RFS: *p* = 3.1e−12), whereas expression of the piCAF subpopulation markers (*MMP1*, *MMP3*, *CXCL8*) does not correlate with survival (OS: *p* = 0.65, RFS: *p* = 0.8). High expression was defined using the upper tertile of the total cohort.

In a recent study, myofibroblasts from different diseased tissues, including pancreatic ductal adenocarcinoma (PDAC), non-small cell lung cancer and COVID-19 lungs, were characterized by increased *CTHRC1* expression and were found to comprise two distinct subpopulations of fibroblasts that were not further specified [14]. Similarly, in human breast cancer, *FAP*-positive CAFs were found to be divided into two subpopulations, termed myCAFs and iCAFs [19, 20]. Using the scRNA-seq dataset from the CRC in vivo cohort, we found that CTHRC1 and FAP are co-expressed in ITGA3-CAFs of CRC (Fig. 6a). Similar to the above observations, ITGA3-CAFs were composed of two subpopulations resembling myCAFs and iCAFs (Fig. 6b). This conclusion is confirmed by the expression of the myCAF markers *ACTA2*, *TAGLN* and *MYL9* in the first subpopulation (Fig. 6c) and by the expression of secreted cytokines (*CXCL1, 3, 8* and *IL6*) characteristic for iCAFs in the second subpopulation (Fig. 6d). Interestingly, in contrast to iCAFs, the second subpopulation also expressed matrix metalloproteinases, such as *matrix metallopeptidase 1* (*MMP1*) and *matrix metallopeptidase 3* (*MMP3*) (Fig. 6c, d). To emphasize this difference, this subpopulation was termed proteolytic inflammatory CAFs (piCAFs). Spearman correlation analysis of both ITGA3-CAF subpopulations revealed that *CXCL8*, *IL6*, *MMP1*, and *MMP3* were highly co-expressed in piCAFs, while *ACTA2*, *TAGLN*, and *MYL9* were found to be strongly correlated in myCAFs (Fig. 6e). Only a weak correlation was observed between the signatures of both CAF subpopulations, suggesting that they represent distinct subpopulations (Fig. 6e). Interestingly, gene signatures of both CAF subtypes were significantly enriched in CSM1 and CMS4 tumors within an independent microarray dataset (GSE39582; *n* = 566 primary colon tumors) (Supplementary Fig. 7a).

In culture myCAFs constituted 96.5% of the CAF population, while piCAFs accounted for less than 4% (Fig. 6f). This was in clear contrast to the equal size of both populations in the CRC tissues (Fig. 6b), suggesting that piCAFs are gradually depleted in culture. This was supported by the bulk RNA-seq analysis, showing that piCAF markers (*MMP1*, *MMP3*, *CXCL8*, and *IL6*) were not differently expressed between cultivated CAFs and NAFs. In contrast, myCAF markers such as *ACTA2* and *TAGLN* were significantly elevated in cultured CAFs (3.16-fold and 1.99-fold, respectively) (Supplementary Table S4). Moreover, all HEX CAF markers except ITGA3 were preferentially associated with the myCAF population (Supplementary Table S5).

The signature of myCAFs was closely associated with “*Epithelial to Mesenchymal transition*”, indicating that myCAFs are migratory and invasively activated (Fig. 6g). Supporting their clinical relevance, the combined myCAF signature comprising *ACTA2*, *TAGLN* and *MYL9* was predictive of poor overall and relapse-free survival in CRC patients (Fig. 6h, left and Supplementary Fig. 7b). In contrast, the piCAF signature (*MMP1*, *MMP3* and *CXCL8*) did not show prognostic value (Fig. 6h, right and Supplementary Fig. 7c).

### MyCAFs and piCAFs can be induced by TNF-α and TGF-β treatment of ADH1B-NAFs

It is not well understood how CAF subpopulations develop from NAFs in CRC. Buechler et al. proposed that PI16-fibroblasts may serve as a resource cell capable of developing into specialized fibroblasts [14]. In line with this hypothesis, we observed that the NAF markers *ADH1B*, *TNXB*, *COL14A1*, *PTGIS*, *CXCL12* and *CFD* were most highly expressed in PI16-NAFs, with their expression decreasing in ADAMDEC1-NAFs and being lost in AGT/TGM2-fibroblasts and ITGA3-CAFs (Table 1).

**Fig. 7:**
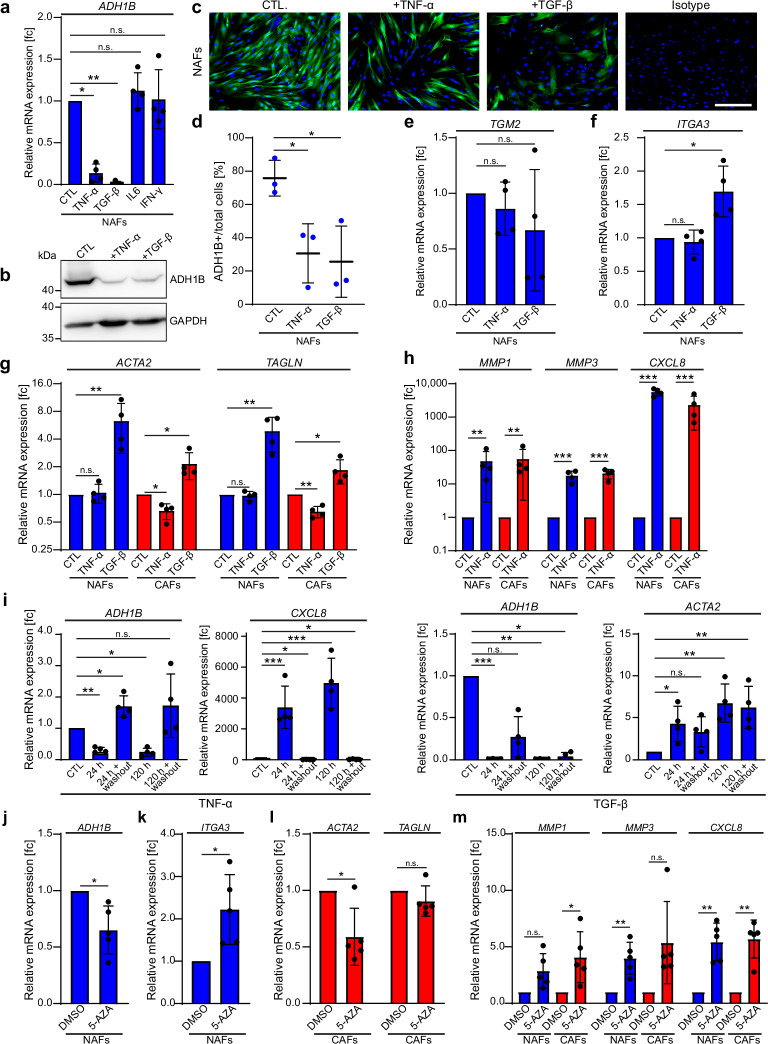
MyCAFs and piCAFs can be induced by TNF-α and TGF-β treatment of ADH1B-NAFs. **a** Impact of TNF-α (50 ng/ml), TGF-β (5 ng/ml), IL-6 (5 ng/ml) and IFN-γ (5 U/ml) on *ADH1B* expression in NAFs (*n* = 4: #3, #8, #12, #14). Gene expression was determined by RT-qPCR and normalized to untreated cells. (Ratio-paired two-tailed *t*-test, n.s. no significance, *P < 0.05, **P < 0.01). **b** Western Blot of ADH1B in NAFs (#12) after repeated TNF-α and TGF-β stimulation. GADPH indicates that equal amounts of protein were loaded. **c** The number of ADH1B positive cells in NAF cultures (*n* = 3: #3, #12, #14) is reduced upon treatment with TNF-α or TGF-β. **d** Quantitative evaluation of the results from (**c**). The total cell count and number of positive cells were determined using ImageJ and averaged among three random areas per sample (unpaired two-tailed *t*-test, *P < 0.05). **e**–**h** Impact of TNF-α and TGF-β on fibroblast subpopulation markers (*TGM2*, *ITGA3*, *ACTA2*, *TAGLN*, *MMP1*, *MMP3* and *CXCL8*) in NAFs and CAFs (*n* = 4 per group: #3, #8, #12, #14). Gene expression was determined by qRT-PCR and normalized to untreated cells. (Ratio-paired two-tailed *t*-test, n.s. no significance, *P < 0.05, **P < 0.01, ***P < 0.001, n.s. no significance). **i** Impact of short-time (24 h) or long-term (120 h) stimulation of NAFs by TNF-α or TGF-β on expression of *ADH1B*, *CXCL8* and *ACTA2*. Cytokines were washed out and gene expression was assessed after 48 h and analyzed by RT-qPCR, normalized to untreated cells (*n* = 4; #3, #7, #9, #10), (Ratio-paired two-tailed *t*-test, n.s. no significance, *P < 0.05, **P < 0.01, ***P < 0.001). **j**–**m** Impact of DNA-Demethylation on *ADH1B*, *ITGA3* expression in NAFs, *ACTA2* and *TAGLN* expression in CAFs and *MMP1*, *MMP3* and *CXCL8* expression in both NAFs and CAFs after 5-Azacitidin treatment. Gene expression was analyzed by RT-qPCR and normalized to DMSO treated cells (*n* = 5 per group; NAFs: #8, #12, #14, #15, #16; CAFs: #8, #12 #14, #15, #17). (Ratio-paired two-tailed *t*-test, n.s. no significance, *P < 0.05, **P < 0.01). #, patient numbers as detailed in Supplementary Tables S1, S2.

These findings prompted us to investigate the potential transition of PI16-NAFs into other fibroblast subpopulations. Recent studies in PDAC demonstrated the induction of myCAFs and iCAFs by TGF-β and IL-1, respectively [21]. In accordance with these findings, we observed deregulation of cytokine-associated pathways, including the TNF-α, TGF-β, IL-6 and IFN-γ pathways, in CAFs, particularly in piCAFs (Fig. 6g), suggesting that the transition of NAFs to CAFs may be related to these cytokines. In agreement with this hypothesis, the expression of the HEX NAF marker *ADH1B* was significantly reduced in NAFs upon stimulation with TNF-α and TGF-β, indicating that both cytokines may contribute to the dedifferentiation and subsequent transition of NAFs into CAFs (Fig. 7a). The cytokines IL-6 and IFN-γ did not affect the expression of *ADH1B* (Fig. 7a). The decrease in *ADH1B* expression induced by TNF-α and TGF-β was confirmed at the protein level (Fig. 7b) and at the single-cell level (Fig. 7c, d). TNF-α and TGF-β did not affect *TGM2* expression in NAFs, indicating that the presence of AGT/TGM2-fibroblasts is not regulated by these cytokines (Fig. 7e).

In a recent review, Waldner et al. reported that the tumor-promoting effects of TGF-β in CRC are mediated by the activation of immune cells and fibroblasts [22]. In agreement with its role in the TME-associated regulation of fibroblast polarization, TGF-β increased the expression of the HEX CAF marker *ITGA3* (Fig. 7f), induced the myCAF marker *ACTA2* and *TAGLN* in NAFs and further elevated the expression of the latter in CAFs (Fig. 7g). In contrast, TNF-α treatment strongly increased the expression of the piCAF marker *CXCL8*, *MMP1* and *MMP3* in both CAFs and NAFs and decreased the expression of *ACTA2* and *TAGLN* in CAFs (Fig. 7g, h). The induction of the piCAF marker *MMP1* in NAFs and CAFs by TNF-α was further confirmed by immunocytochemistry (Supplementary Fig. 8a). Moreover, gelatin zymography and increased collagen contraction confirmed that TNF-α increased the ECM remodeling activity of fibroblasts through the secretion of MMPs (Supplementary Fig. 8b, c).

Single-cell sequencing of cultured CAFs indicated that only myCAFs represent a stable CAF phenotype (Fig. 6f). To validate this, NAFs were exposed to TNF-α or TGF-β for short (24 h) or chronic (120 h) stimulation to induce the respective CAF subtypes, followed by cytokine washout and gene expression analysis after 48 h. Strikingly, *ADH1B* expression was reconstituted after removal of TNF-α (Fig. 7i, left), while TGF-β caused a permanent loss after chronic stimulation (Fig. 7i, right). Accordingly, CXCL8 expression did not persist after washout of TNF-α, independent of stimulation duration, while ACTA2 expression remained stably elevated after chronic TGF-β stimulation (Fig. 7i, right).

Recently, cultivated tumor vessel endothelial cells from CRC were shown to stably maintain different transcriptomes to matching normal colon endothelial cells, which were manifested by microenvironment-dependent epigenetic imprinting [23]. In agreement with this report, we found that the transcriptomic and functional differences between CAFs and NAFs were stably maintained in culture (Fig. 1a–d). Accordingly, we investigated the impact of DNA methylation on the maintenance of different fibroblast subpopulations. To achieve this goal, CAFs and NAFs were cultivated for 9 days in full medium with daily treatment with 10 μM 5-Aza-2´-desoxycytidin (5-Aza) to suppress DNA methylation. Under these conditions, *ADH1B*, was downregulated, whereas *ITGA3* was significantly upregulated in NAFs (Fig. 7j–k). These findings indicate that a decreased methylation status, which is commonly associated with tumor tissues [24], may promote the transition from NAFs to CAFs. The inhibition of methylation preferentially induced piCAF markers (*MMP1*, *MMP3* and *CXCL8*), whereas *ACTA2* and *TAGLN* were mildly reduced (Fig. 7l, m). These findings demonstrated that reduced methylation leads to the formation of piCAFs from both NAFs and myCAFs, which provides perspectives for modulation of CAF phenotypes.

## Discussion

Here we showed that ADH1B and ITGA3 can sensitively distinguish the total NAF population from CAFs in CRC patients, and are easily applicable for immunofluorescence or histochemical studies at the single-cell level in culture and tissues. However, ITGA3 is not fibroblast-specific and is also expressed by epithelial cells. Consequently, the utilization of ITGA3 in conjunction with mesenchymal and epithelial exclusion markers is advocated for the precise identification of CAFs.

In agreement with previous findings we found that CAFs in CRC were composed of two populations: myCAFs and a population resembling previously described inflammatory CAFs (iCAFs) [25], which was described in PDAC, breast and rectal cancer [4, 20, 25, 26]. Interestingly, the iCAFs detected here, showed increased upregulation of metalloproteases. To reflect this feature, which may arise from regulatory differences in the tumor microenvironment of CRC, we refined this population as proteolytic inflammatory CAFs (piCAFs). Although metalloprotease expression suggests potential involvement in ECM remodeling, the impact of piCAF-derived MMPs on CRC progression, therapy, or metastasis remains to be established. Additionally, piCAF marker expression was not associated with prognosis, which motivates further investigation into whether shifts between myCAF and piCAF phenotypes have functional or therapeutic relevance in colorectal cancer.

In contrast to CAFs, the heterogeneity of NAFs has been sparsely investigated. PI16^+^ cells were previously suggested to establish a resource fibroblast population present in normal tissues that may develop into specialized fibroblasts [14]. *ADAMDEC1-*fibroblasts have been associated with myofibroblast characteristics in colitis. [14, 27]. In this study, we found that PI16- and ADAMDEC1-NAFs together establish the ADH1B-NAF population in the normal colon. We show that NAFs loose ADH1B expression during transdifferentiation to CAFs, which may warrant to investigate the role of ADH1B in this process.

In addition, AGT/TGM2-fibroblasts were identified as a novel subpopulation, distinct from both ADH1B-NAFs and ITGA3-CAFs. The transcriptomes of these cells indicated putative functions in ECM organization. TGM2 is a transglutaminase that can be secreted and is involved in many biological processes, including wound healing and the ligation of extracellular matrix molecules [28, 29]. In CRC, TGM2 expression in tumor cells is associated with a poor prognosis and resistance to chemotherapy [30, 31]. In addition, angiotensin can stimulate the proliferation of CRC cells and fibroblasts [32]. Both observations suggest an active role of AGT/TGM2-fibroblasts in CRC pathogenesis, but the function of both markers in fibroblasts is currently unknown. The specific functional contribution of AGT/TGM2- fibroblasts in CRC remains therefore unclear and warrants targeted investigation. Of note, AGT/TGM2-fibroblasts coexists in the colons of both healthy and CRC patients and may establish a transition phenotype, thus challenging the existing dogma of fibroblast categorization into either NAFs or CAFs.

Importantly, fibroblast subpopulations are predominantly defined by bioinformatic analyses of one-point scRNA-seq data directly from tissues. This approach does not provide information about the long-term stability of the different phenotypes under culture conditions. To address this gap, we compared the subpopulations of NAFs and CAFs maintained in culture. Importantly, PI16-NAFs, AGT/TGM2-fibroblasts and myCAFs were stably maintained in culture, whereas ADAMDEC1-NAFs and piCAFs were detected only in analyses of tissues. These findings indicate that the phenotypes of ADAMDEC1-NAFs and piCAFs are transiently induced in vivo and are lost during isolation.

The final questions addressed which factor may induce the formation of CAF subtypes and whether these fibroblast states exhibit plasticity toward other phenotypic programs. TGF-β is a likely candidate for CAF induction, because its expression is associated with a worse prognosis in CRC, and numerous studies have demonstrated its effects on fibroblast activation and worse outcomes in various cancers [21, 33–36]. In fact, TGF-β treatment induced the differentiation of NAFs into myCAFs These findings are consistent with the report that PI16-NAFs establish an unspecialized universal resource cell, which may be the primary provider of myCAFs [14].

On the other hand, the TNF pathway counteracts tumorigenesis and is involved in the induction of cell death in CRC [37–39] and suppresses the expression of α-SMA in human dermal fibroblasts [40]. In our experiments, TNF-α treatment reduced expression of canonical myCAF markers and promoted a piCAF-like transcriptional profile. Moreover, it has been reported that a pro-invasive CAF phenotype from breast cancer manifests via epigenetic mechanisms [41]. These observations indicate that epigenetic imprinting may stabilize different subpopulations of NAFs and CAFs. This hypothesis was clearly supported by the finding that treatment of PI16-NAFs and myCAFs with the DNA-demethylating agent 5-Aza converted them to transient piCAFs. Likewise, it was demonstrated that chronic TGF-β exposure induces DNA-methylation in fibroblasts [42].

In summary, new NAF and CAF differentiation markers were identified and a map of all major fibroblast subpopulations in the colon of CRC patients was established. The presence of the different populations was confirmed at the single-cell level in culture and tissues, and external cues associated with shifts between normal fibroblasts, myCAFs, and piCAFs were characterized. Our findings offer new perspectives for characterizing the functions and dynamic plasticity of fibroblast populations during the development and treatment of colorectal cancer, as well as new targets and approaches for stromal cell-directed therapy.

## Supplementary information

SUPPLEMENTAL MATERIAL

## Data Availability

This study used cultures of NAFs and CAFs isolated in previous work as described. The extended methods are provided in the supplementary information. Bulk RNA-seq (E-MTAB-14208) and scRNA-seq (E-MTAB-14227) data have been deposited at ArrayExpress and are publicly available as of the date of publication. Any additional information required to reanalyze the data reported in this paper is available from the lead contact upon request.
