# Supplementary material for: Fibroblast dynamics in colorectal cancer: stability, plasticity, and novel markers
Source: Oncogene. 2026 Apr 28;45(24):2299–312. doi: 10.1038/s41388-026-03809-6 (PMC13249576; doi:10.1038/s41388-026-03809-6)
Supplement: Supplementary file 1 — SUPPLEMENTAL MATERIAL [file 41388_2026_3809_MOESM1_ESM.pdf]

Fibroblast Dynamics in Colorectal Cancer: Stability, Plasticity, and Novel Markers

Richard Demmler[1,2,3], Charles G. Anchang[2,3,4], Yongsong Yong[1], Andreas Ramming[2,3], Simon Rauber[2,3], Vera S. Schellerer[1,5], Benjamin Schmid[6], Arndt Hartmann[7,8,9,10], Susanne Merkel[8,11], Katharina Imkeller[4,12,13], Elisabeth Naschberger[1,8,9,10], Michael Stürzl[1,8,9,10*]

[1] Division of Molecular and Experimental Surgery, Uniklinikum Erlangen, Friedrich-Alexander-Universität Erlangen-Nürnberg (FAU), Erlangen, Germany

[2] Department of Medicine 3 - Rheumatology and Immunology, Friedrich-Alexander-Universität Erlangen-Nürnberg and Uniklinikum Erlangen, Erlangen, Germany

[3] Deutsches Zentrum für Immuntherapie (DZI), Friedrich-Alexander-Universität Erlangen-Nürnberg and Uniklinikum Erlangen

[4] Institute of Neurology (Edinger Institute), Goethe University, University Hospital Frankfurt, Frankfurt/Main, Germany

[5] Present address: Department of Pediatric Surgery, University of Greifswald, Greifswald, Germany

[6] FAU Competence Centre Optical Imaging Centre Erlangen, Friedrich-Alexander-Universität Erlangen-Nürnberg (FAU), Erlangen, Germany

[7] Institute of Pathology, Uniklinikum Erlangen, Friedrich-Alexander-Universität Erlangen-Nürnberg (FAU), Erlangen, Germany

[8] CCC Erlangen-EMN: Comprehensive Cancer Center Erlangen-EMN (CCC ER-EMN), Erlangen, Germany

[9] CCC WERA: Comprehensive Cancer Center Alliance WERA (CCC WERA), Erlangen, Germany

[10] BZKF: Bavarian Cancer Research Center (BZKF), Erlangen, Germany

[11] Department of Surgery, Uniklinikum Erlangen, Friedrich-Alexander-Universität Erlangen-Nürnberg (FAU), Erlangen, Germany

[12] University Cancer Center (UCT), Frankfurt/Main, Germany

[13] Frankfurt Cancer Institute (FCI), Frankfurt/Main, Germany

* Corresponding author

Supplemental Figure 1

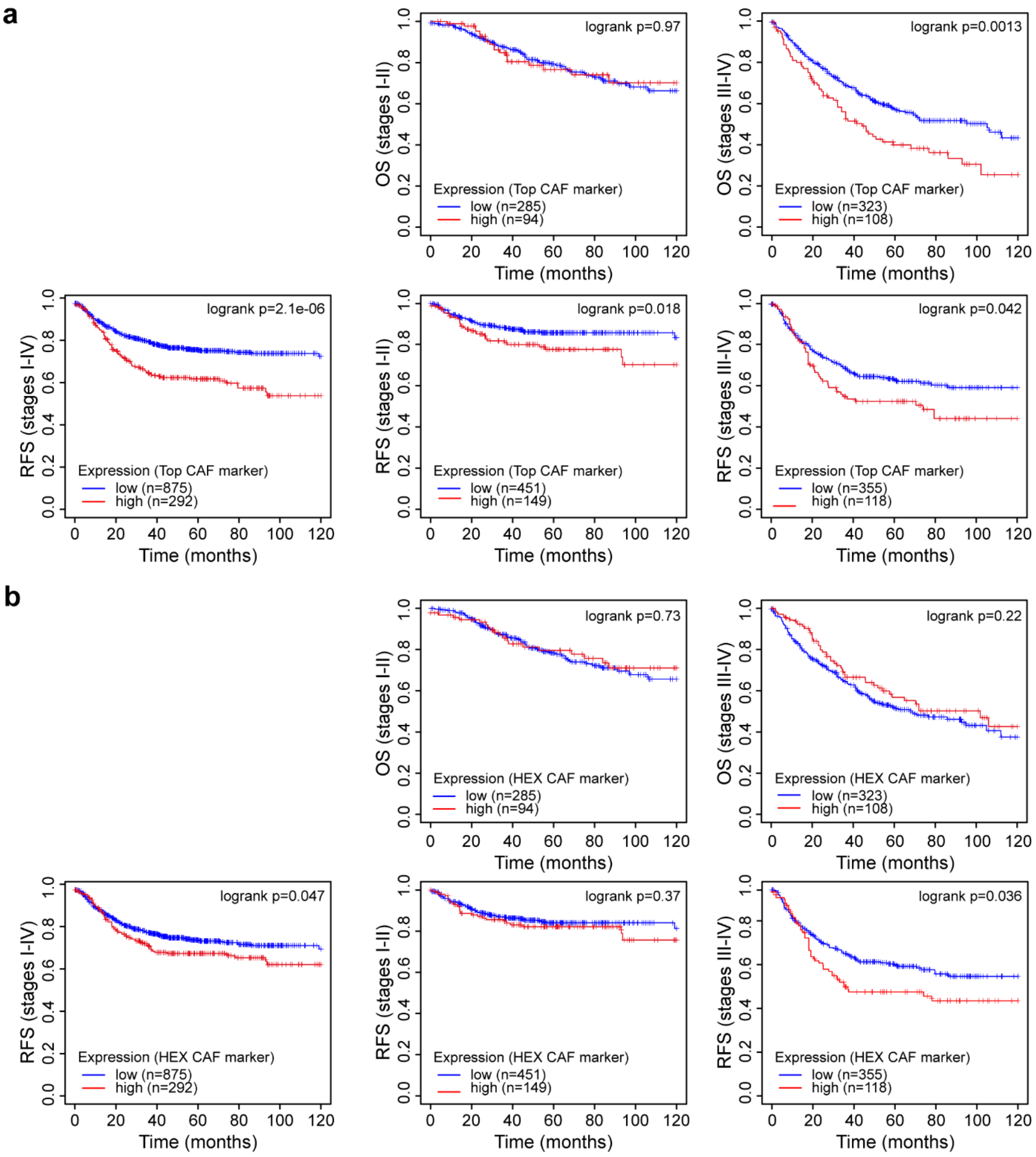

Supplemental Figure 1: Prognostic impact of TOP CAF and HEX CAF markers in CRC. Kaplan–Meier analyses assessing overall survival (OS) and relapse-free survival (RFS) according to high versus low expression of **(a)** the TOP CAF markers and **(b)** HEX CAF markers. Plots are stratified by clinical stage (I–II, III–IV, and all stages combined). High expression was defined using the upper quartile of the total cohort.

Supplemental Figure 2

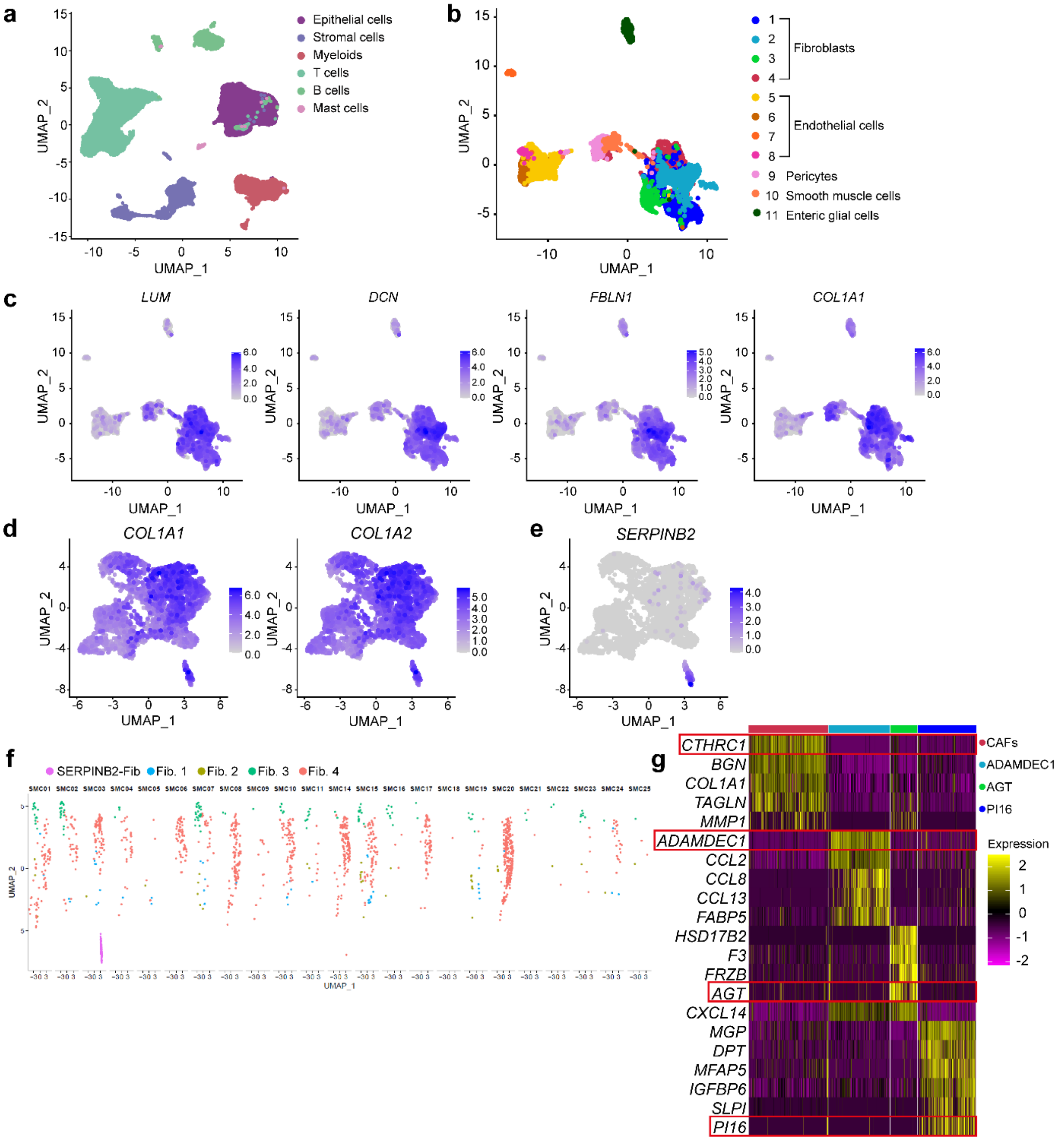

Supplemental Figure 2: Identification of fibroblast subpopulations in normal colon and CRC tissues.
(a) and **(b)** UMAP-clustering of all cells captured in the *in vivo* cohort by Lee et al. was conducted (23 CRC and 10 matching normal colon tissues) [1]. Stromal cells clustered together, separated from epithelial, mast, myeloid and immune cells. **(c)** A combination of *LUM*, *DCN*, *FBLN1* and *COL1A1* was used to discriminate fibroblasts from other stromal cells. **(d)** UMAP plot depicting gene expression of *COL1A1* and *COL1A2* among all fibroblasts captured in the dataset. **(e)** *SERPINB2* expression is restricted to a small subgroup of fibroblasts. **(f)** Distribution of fibroblast subpopulations among all analyzed patients. A *SERPINB2* expressing cluster was only present in the CRC tissue of a single patient and was removed as outlier. All other subpopulations are represented in the majority of the patients. **(g)** Heat map representing the genes characterizing distinct fibroblast subpopulations in healthy colon and CRC.

Supplemental Figure 3

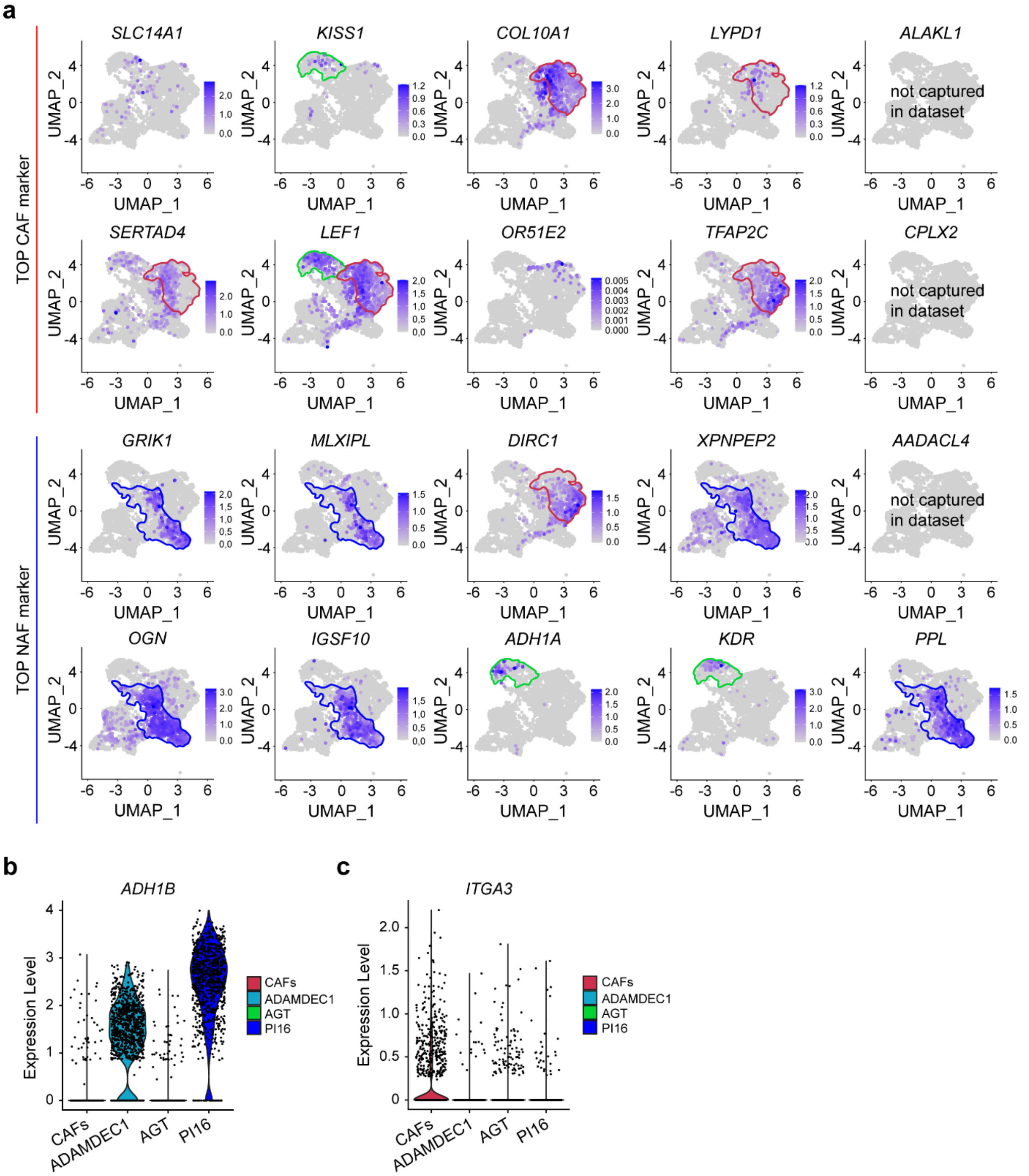

Supplemental Figure 3: Top CAF and NAF differentiation markers are associated with distinct fibroblasts subpopulations.
(a) UMAP plots displaying the expression levels of the top CAF and NAF differentiation markers by log2fc in the fibroblast subpopulations identified from the published *in vivo* scRNA-seq dataset [1]. The subpopulations with the highest relative expression are circled (PI16-NAFs: dark blue, ADAMDEC1-NAFs: light blue, AGT-fibroblasts: green, CAFs: red). The top NAF differentiation markers are associated with the PI16-subpopulation (dark blue), whereas the top CAF differentiation markers are expressed by CAFs (red) and AGT-fibroblasts (green). **(b and c)** Expression of the HEX markers *ADH1B* and *ITGA3* among the four identified subpopulations of fibroblasts.

Supplemental Figure 4

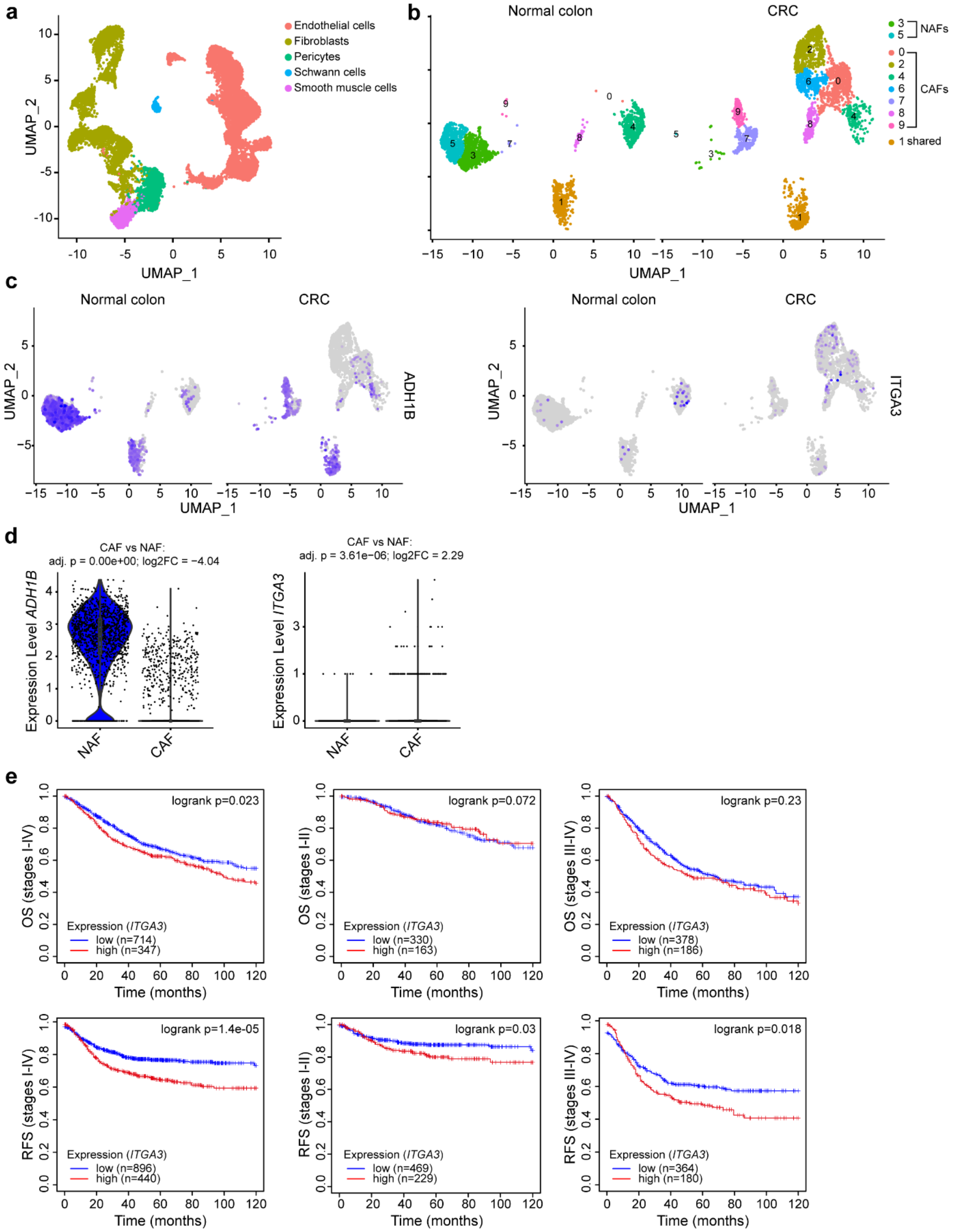

Supplemental Figure 4: Validation of ADH1B and ITGA3 expression in NAFs and CAFs.
(a) UMAP-clustering of all cells captured in the cohort by Pelka et al. [2] was conducted on 267,489 cells retained after filtering, of which 68,630 were from healthy tissue and 198,859 from tumor tissue. **(b)** Fibroblasts cells clustered together, separated by tissue origin **(c)** UMAP plot depicting gene expression of *ADH1B* and *ITGA3* among all fibroblasts captured in the dataset. **(d)** *ADH1B* and *ITGA3* expression in NAFs and CAFs **(e)** Kaplan–Meier analyses assessing overall survival (OS) and relapse-free survival (RFS) according to high versus low expression of *ITGA3*. Plots are stratified by clinical stage (I–II, III–IV, and all stages combined). High expression was defined using the upper tertile of the total cohort.

Supplemental Figure 5

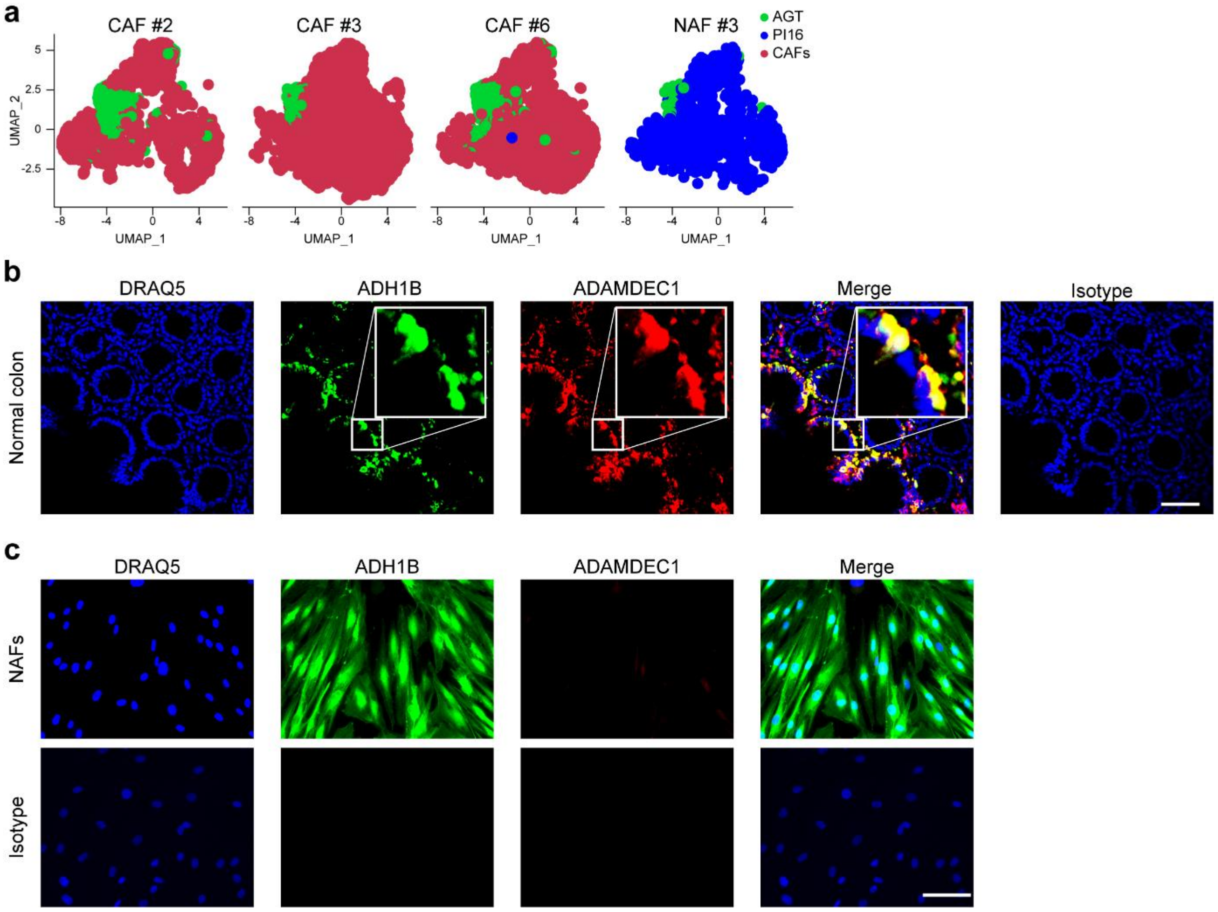

Supplemental Figure 5: ADAMDEC1-NAFs are depleted in long-term *in vitro* cultures.
ScRNA-seq analyses of fibroblasts cultivated from CRC (n = 3; #2, #3, #6) and normal colon (n = 1; #3) detects presence of AGT-fibroblasts and the CAF-phenotype in all three CAF cultures. PI16-NAFs are exclusively detected in the NAF sample, while ADAMDEC1-NAFs are depleted. **(b)** ADAMDEC1^{+}ADH1B^{+} cells are detectable *in vivo* (Scalebar represents 50 µm), **(c)** but are depleted in the initial outgrowth of non-passaged NAF cultures (NAF #3, Scalebar represents 100 µm). #, patient numbers as detailed in tables S1 and S2.

Supplemental Figure 6

a

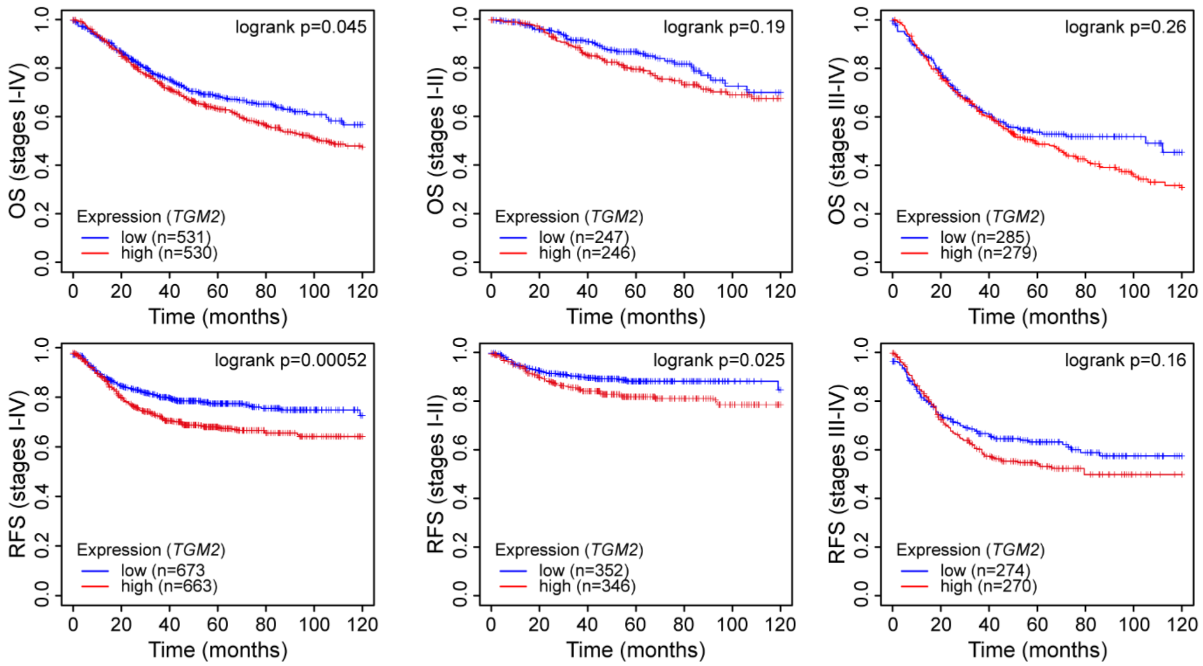

Supplemental Figure 6: Prognostic impact of TGM2 in CRC.
(a) Kaplan–Meier analyses assessing overall survival (OS) and relapse-free survival (RFS) according to high versus low expression of *TGM2*. Plots are stratified by clinical stage (I–II, III–IV, and all stages combined). Patients were stratified by median expression of the total cohort.

Supplemental Figure 7

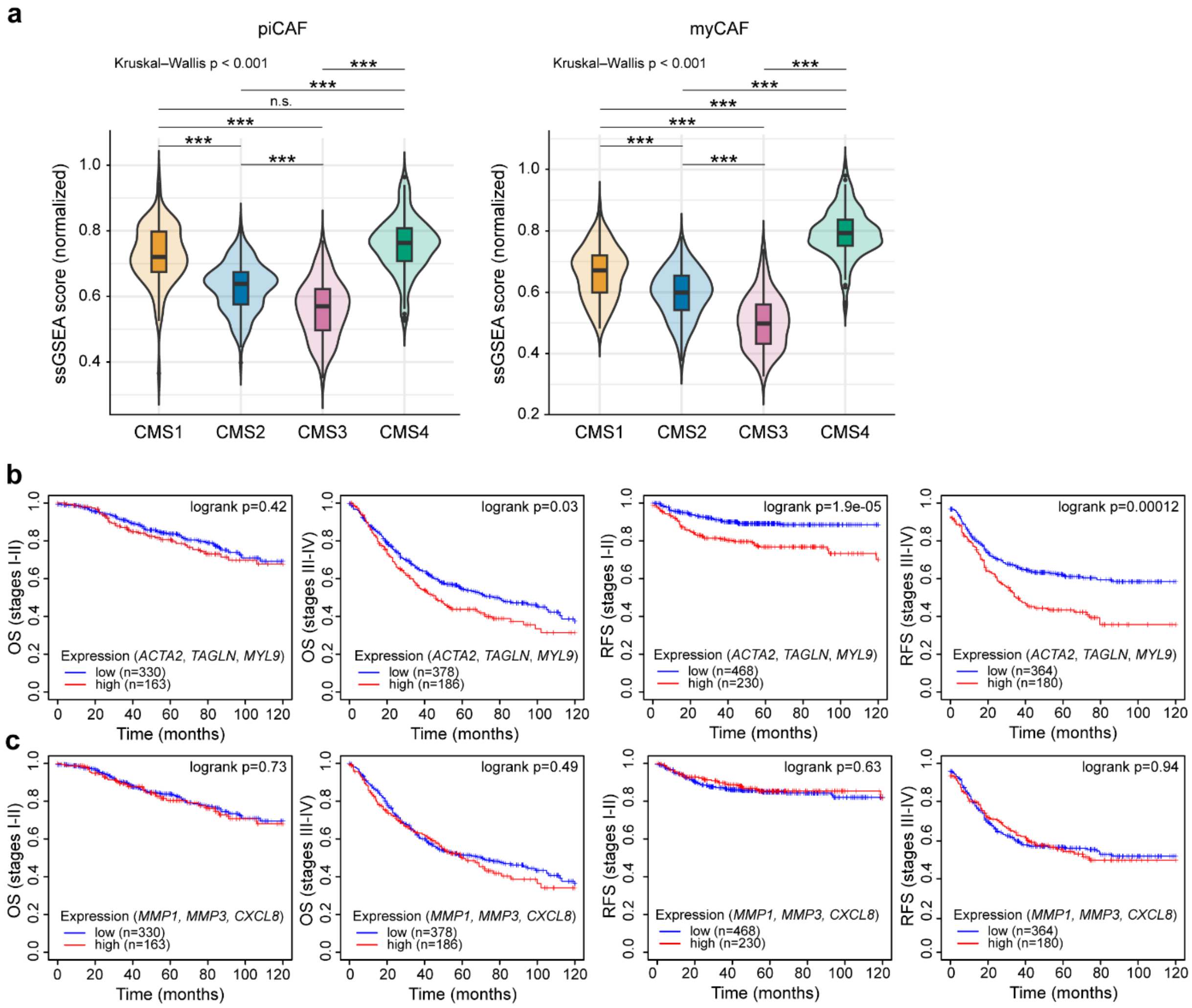

Supplemental Figure 7: Clinical association of CAF subtypes in CRC.
(a) Gene signatures of piCAFs and myCAFs among CMS subclasses in GSE39582. Global p-value was determine using the Kruskal–Wallis rank-sum test. Pairwise comparisons were conducted by Post-hoc Dunn tests (BH-adjusted, n.s.= no significance, ****P* <0.001). **(b-c)** High expression of myCAF markers (*ACTA2*, *TAGLN*, *MYL9*) is associated with worse OS and RFS for CRC patients in both early and late-stage patients, whereas expression of the piCAF subpopulation markers (*MMP1*, *MMP3*, *CXCL8*) does not correlate with survival. High expression was defined using the upper tertile of the total cohort.

Supplemental Figure 8

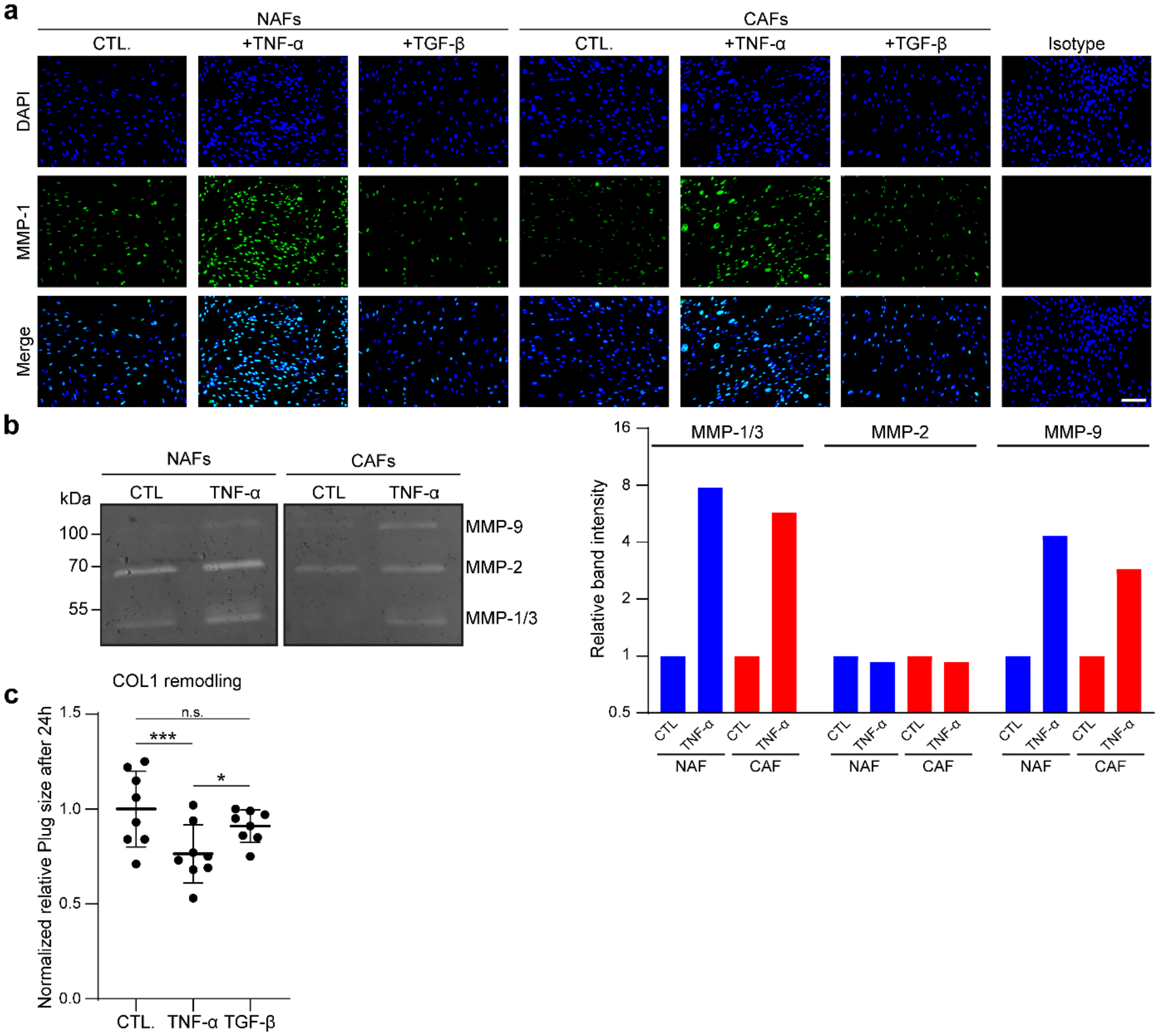

Supplemental Figure 8: Proteolytic inflammatory CAFs are engaged in ECM remodelling by release of MMP-1 and MMP-3.
(a) Increased levels of MMP1 can be observed upon TNF-α stimulation in both NAFs and CAFs as shown by immunocytochemistry staining (NAF#12, CAF#11). TGF-β stimulation did not alter MMP1 expression. Scalebar represents 150 µm. **(b)** Gelatin-Zymography confirms the release of active MMP-1, MMP-3 and MMP-9 from NAFs (#8, blue) and CAFs (#12, red) after TNF-α but not TGF-β treatment. MMP-2 levels were not affected (left). The band intensities were displayed using the "plot lane" tool in ImageJ and the area under the curve was quantified after the background signal was removed (right). **(c)** Normalized relative collagen 1 plug size after 24 h upon fibroblast treatment with TNF-α or TGF-β stimulation (n=8, NAFs: #2, #3, #12, #14; CAFs: #2, #3, #8, #12). (Paired two-tailed *t-test,* n.s.= no significance, **P* <0.05, ****P* <0.001). #, patient numbers as detailed in tables S1 and S2.

Table S1: Clinical characteristics of the colorectal carcinoma patients from which fibroblasts were isolated.

Patient	Sex	Age	UICC stage	Localization	T	N	M	L	V
1	male	72	IV	Coecum	4b	1b	1	0	0
2	female	61	IIIC	Sigma	4a	2b	1	1	0
3	male	59	IIIB	Sigma	3	1b	0	0	0
4	female	68	IIA	Sigma	3	0	0	0	0
5	female	51	IIA	Transversum	3	0	0	0	0
6	male	55	IB	Rectum	2	0	1	1	1
7	female	75	IIA	Sigma	3	0	0	0	0
8	male	80	N/A	Coecum	NA	NA	1	1	0
9	female	89	IIA	Colon ascendens	3	0	0	0	0
10	female	71	IIIA	Rectum	1	1b	1	0	0
11	male	68	IIA	Flexura lienalis	3	0	0	0	0
12	male	69	IB	Rectum	2	0	1	0	0
13	female	80	III	Coecum	3	2b	1	0	1
14	female	58	IVB	Sigma	NA	NA	1	1	0
15	male	51	IIA	Rectum	3	0	0	0	0
16	male	72	IIB	Rectum	4a	0	1	0	0
17	male	75	IIA	Coecum	3	0	0	0	0

Sex, age, UICC stage, tumor localization, T-, N-, M-categories and L-, V-classification are given for each patient (N/A: information not available). Usage of respective NAF and CAF cultures in the different experiments is depicted in table S2 and in the figure legends.

Table S2: Experimental use of respective NAF and CAF cultures in this study.

Patient	Bulk RNA-seq		scRNA-seq		Immuno-cytochemistry		COL1-assay (unstimulated)		5-Aza treatment		Cytokine treatment	
	NAF	CAF	NAF	CAF	NAF	CAF	NAF	CAF	NAF	CAF	NAF	CAF
**1**	-	+	-	-	-	-	-	-	-	-	-	-
**2**	+	+	-	+	+	+	-	-	-	-	-	-
**3**	+	+	+	+	+	+	+	+	-	-	+	+
**4**	+	+	-	-	-	-	-	-	-	-	-	-
**5**	-	+	-	-	-	-	-	-	-	-	-	-
**6**	-	+	-	+	-	-	-	-	-	-	-	-
**7**	-	-	-	-	-	-	+	+	-	-	+	-
**8**	-	-	-	-	+	+	+	+	+	+	+	+
**9**	-	-	-	-	-	-	-	-	-	-	+	-
**10**	-	-	-	-	-	-	-	-	-	-	-	-
**11**	-	-	-	-	+	+	+	+	-	-	-	-
**12**	-	-	-	-	+	+	+	+	+	+	+	+
**13**	-	-	-	-	-	-	-	-	-	-	+	-
**14**	-	-	-	-	+	+	-	-	+	+	+	+
**15**	-	-	-	-	-	-	-	-	+	+	-	-
**16**	-	-	-	-	-	-	-	-	+	-	-	-
**17**	-	-	-	-	-	-	-	-	-	+	-	-

Table S3: Differentially expressed genes in cultured primary CAFs (n=6) compared to NAFs (n=3) based on bulk RNA-seq.

	Gene	log2FC CAF vs. NAF	P-adj	Normalized counts CAFs	Normalized counts NAFs
**Top CAF marker**	*SLC14A1*	6.07	9.86E-16	413.51	3.98
	KISS1	5.98	2.75E-10	553.65	4.06
	COL10A1	5.92	1.50E-10	222.91	1.82
	LYPD1	5.81	1.18E-19	1877.44	24.91
	ALKAL1	5.76	1.08E-34	997.30	15.89
	SERTAD4	5.65	5.38E-12	189.43	2.38
	LEF1	5.64	2.06E-07	316.37	2.05
	OR51E2	5.52	7.83E-07	180.34	1.00
	TFAP2C	5.23	5.25E-15	348.91	6.88
	CPLX2	5.16	2.16E-07	93.88	1.08
**Highly expressed CAF marker** (CAF counts >5000)	*CXCL14*	2.94	4.28E-04	6112.57	256.40
	SORT1	2.55	2.67E-10	7218.84	1051.63
	AMIGO2	2.42	5.55E-09	5024.34	797.69
	RRM2	2.27	1.08E-06	6740.54	1131.19
	WFDC1	2.25	1.65E-08	14938.81	2710.81
	GAS6	2.22	3.49E-09	23791.38	4483.47
	ITGA3	2.10	2.72E-06	10031.58	1937.45
	MCAM	2.10	3.28E-19	22347.28	4964.62
	ANLN	2.08	3.21E-07	9106.19	1853.21
	GFRA1	2.04	1.90E-03	9378.23	1047.13
**Top NAF marker**	*GRIK1*	-6.51	3.03E-20	1.78	251.37
	MLXIPL	-6.16	6.45E-15	1.95	226.21
	DIRC1	-5.79	2.82E-09	0.39	43.42
	XPNPEP2	-5.75	1.55E-23	3.82	265.08
	AADACL4	-5.43	2.67E-08	0.33	34.01
	OGN	-5.34	9.94E-07	2.00	275.64
	IGSF10	-5.29	4.37E-23	15.16	721.73
	ADH1A	-5.25	6.64E-12	3.02	174.19
	KDR	-4.88	5.34E-12	1.85	74.13
	PPL	-4.69	1.18E-19	66.07	2013.37
**Highly expressed NAF markers** (NAF counts >5000)	*ADH1B*	-3.79	1.27E-04	498.26	30272.94
	SELENOP	-3.74	5.94E-22	419.72	6239.13
	TNXB	-3.64	2.93E-10	2170.42	35301.14
	COL14A1	-3.45	2.05E-11	3993.94	54265.38
	PTGIS	-3.27	7.07E-06	2044.91	33648.27
	FAM167A	-3.17	3.91E-13	625.60	6656.09
	CXCL12	-3.17	1.81E-29	1866.23	17946.63
	FBLN2	-2.84	1.38E-10	1894.15	16309.37
	CFD	-2.77	2.14E-06	625.27	6033.72
	CYP1B1	-2.72	3.03E-09	1189.75	9601.57

Table S4: Differential gene expression of myCAF and piCAF markers in cultured CAFs (n=6) compared to NAFs (n=3).

	Gene	log2FC CAF vs. NAF	P-adj
**myCAF**	*ACTA2*	1.66	6.37E-06
	TAGLN	0.99	0.00044
**piCAF**	*MMP1*	0.18	0.4865
	MMP3	0.22	0.4207
	CXCL8	-0.12	0.6784

Table S5: Differential gene expression of the HEX CAF markers between myCAFs and piCAFs.

Gene	P	log2FC	P-adj	cluster
CXCL14	0.00026	0.38	> 0.05	myCAF
SORT1	1.55E-40	1.38	2.51E-36	myCAF
AMIGO2	1.04E-81	2.55	1.68E-77	myCAF
RRM2	4.79E-06	1.87	0.077	myCAF
WFDC1	1.81E-23	1.97	2.94E-19	myCAF
GAS6	3.17E-19	0.83	5.13E-15	myCAF
MCAM	3.59E-17	1.80	5.81E-13	myCAF
ANLN	0.00023	1.64	> 0.05	myCAF
GFRA1	8.54E-05	2.18	> 0.05	myCAF

Table S6: Primer sequences used for RT-qPCR.

Target	5' - 3' forward Primer	5' - 3' Reverse Primer
ADH1B	GGGGCTGTTTATGGTGGCTTT	ACTGCTACAGGGGAAGGCAT
SELENOP	GGAAACTGCTCTCTCACGACTCT	TGGTGCTCCTGGTTGCTGAT
TNXB	ACTGTGCCATCCAGACATGC	AGTCTTCGCCAGCATACCCA
COL14A1	ACCAGGGGTGGAAAACCAGA	AGCCATCGGTAAAGCAAGTGT
PTGIS	ACCACTCTCCCACAGAAGGT	TCACTCAGCACGCTATCAAGC
FAM176A	CTCAGGAAGGAACTGACGGAGAT	AGCTCGTAGGTGGCATCGTT
CXCL12	CTGTGCCCTTCAGATTGTAGCC	TCGAGTGGGTCTAGCGGAAA
FBLN2	TCTCCTGCTGTGAGGGTGAA	TCTCTGCCTCTGAAACTCTCCG
CFD	AAGCAACAAAGTCCCGAGCAA	AAGACCAACCAGATGCAGGAGT
CYP1B1	ACGTACCGGCCACTATCACT	ACCCATACAAGGCAGACGGT
CXCL14	AATGAAGCCAAAGTACCCGCA	GACACGCTCTTGGTGGTGAT
SORT1	TTCCAGGGGACAAATGCCAG	AGCATCAATCCCACGATGGC
AMIGO2	TTCCCAGCGAGGCAGTGATA	ACGCCACAAAAGGTGTGTCA
RRM2	CCCACGGAGCCGAAAACTAA	TGGGGAAGATGACAAAGCGG
WFDC1	TACGCCTGCCTAGAAGCTGT	CTGCACGCCTCTGCTTGTAA
GAS6	ACGACCCCGAGACGGATTAT	GCACTGGTCAGGCAGGTTTT
ITGA3	CTCACCCCTCACTCCTTCTTCA	TTACCTGCTGGGGCTGTCTA
MCAM	AACATCCAGGTCAACCCCCT	ACCACTCGACTCCACAGTCT
ANLN	GGATGGCGATGCCTCTTTGA	ACCCCCGTTTTCAGAGGACT
GFRA1	TTCCGGGTGGTCCCATTCAT	TGGGAATGTGCTCCACTTGC
TGM2	TGGTGAGTGGCATGGTCAAC	TTGTAGTTGGTCACGACGCG
ACTA2	GTGGGTGACGAAGCACAGAG	GGGGCAACACGAAGCTCATT
TAGLN	ATGATGGGCACTACCGTGGA	GCCCTCTCCGCTCTAACTGA
MMP1	CGCTGGGAGCAAACACATCT	TTGTCCCGATGATCTCCCCT
MMP3	TCCCTCCAACCGTGAGGAAA	GCTATTTGCTTGGGAAAGCCTG
CXCL8	GGAGAAGTTTTTGAAGAGGGCTGA	ACTGGCATCTTCACTGATTCTTGG
ADAMDEC1	TGTGTTCCCCATCTTCGGTTG	AGGCATACCAAGGACATGGC
CCL2	ACCTTCATTCCCCAAGGGCT	GGTTTGCTTGTCCAGGTGGT
CCL8	CATGCTGAAGCTCACACCCT	ATCCCTGACCCATCTCTCCTTG
RPL37A	TGTGGTTCCTGCATGAAGACA	GTGACAGCGGAAGTGGTATTGTAC

Extended Methods

Cytokine stimulation

Prior to cytokine stimulation, the cells were grown until 80-90% confluence under standard culture conditions, followed by starvation for 24 h in low medium (0.5% FCS). Stimulation was conducted with either 5 U/ml IFN-γ (Roche, Basel, Switzerland; #11040596001), 5 ng/ml IL-6 (Roche, #11138600001), 50 ng/ml TNF-α (Roche, #11371843001) or 5 ng/ml TGF-β (R&D Systems, Minneapolis, Minnesota; #7754-BH-005/CF) or 1 µg/ml LPS (Invitrogen, #00-4976-93) in low medium for 24 h (short-term) or 120 h (chronic).

DNA methylation test

The impact of DNA methylation on fibroblast subpopulation marker expression was addressed by daily exposure to 10 µM 5-Aza-2´-Desoxycytidin (5-Aza) (Sigma–Aldrich, #A3656) for 9 days until RNA was extracted.

RNA extraction

Cells were harvested via trypsinization and subsequent centrifugation. The cell pellet was washed with 1x PBS, lysed in cold RLT buffer with 1% ß-mercaptoethanol (Merck, Darmstadt, Germany; #M6250) and passed through a QIAshredder column (Qiagen, Venlo, Netherlands; #79654). The lysate was processed using the RNeasy Mini Kit (Qiagen, #74104) according to the manufacturer´s instructions, followed by purification with 0.108 µg/µl glycogen (Thermo Fisher, #R0551). Finally, pure RNA was resuspended in RNase-free water and analyzed for quantity and integrity using a Nanodrop 2000 (Thermo Fisher).

Reverse transcription qPCR

RT-qPCR was performed with SYBR Assay ROX (Eurogentec, Seraing, Belgium; #RT-SN2X-03+) as previously described [3]. The primers used were obtained from Eurofins Scientific (Luxemburg, Luxemburg) and are listed in Table S6.

Collagen-1 remodeling test

The ability of NAFs and CAFs to remodel a collagen 1 matrix was evaluated as described previously [4]. In brief, 50,000 cells were seeded in duplicate into a rat tail collagen 1 plug at a final concentration of 1.8 mg/ml (Corning, NY, USA; #354249), adjusted with sterile NaOH and 10x PBS to pH 7. If needed, cytokines were included in both the plug and the overlaying medium. The shrinkage of the plug size was determined after 24 hours and measured using ImageJ.

Bulk RNA sequencing of cultured human fibroblasts

Total RNA of CAFs (n=6) and NAFs (n=3) (all passage <2) was isolated as described. A greater number of CAFs were included, as it was anticipated that this group would exhibit greater variability. Sequencing was performed in 100 bp 3´ single-end mode on a HiSeq2500 (Illumina, San Diego, California, USA) at >30 million reads per sample. Normalization and differential expression analyses were performed using the default parameters of the counts and DSEq functions in the DESeq2 R package version 1.22 [5] with Wald test and adjusted p value <0.05 as threshold. DEGs with fold change >1.5 were used for Reactome pathway enrichment (ReactomePA v1.26.0) [6]. HEX markers were defined by applying a threshold of 5 000 mean reads on protein-coding genes. Marker-based decomposition was conducted to estimate the proportions of the fibroblast subpopulations in NAFs and CAFs in the bulk RNA-seq dataset. Subpopulation proportions in the bulk data were estimated using MarkerBasedDecomposition

(BisqueRNA package) without weighting [7] with marker input derived from cluster-based enrichment.

Single-cell RNA sequencing

Single-cell RNA-seq was conducted using the Chromium Next GEM Single Cell 3' Kit v3.1 (#1000269) and the Chromium Next GEM Chip G Single Cell Kit (#1000127) from 10X Genomics (Pleasanton, California, USA). Cells of 3 CAF cultures and one NAF culture (passage 1–2.5) were processed according to the manufacturer's instructions. Library construction was conducted with the library construction kit (#1000196) followed by indexing with the Dual Index Kit TT Set A (#3000431). Sequencing was performed by Novogene Co. (Cambridge, UK) on a NovaSeq 6000 (Illumina) in 150 bp, paired-end mode at >300 million reads/sample.

<u>Bioinformatic analysis:</u> The reads were aligned to the human reference genome (GRCh38) using the count command from Cell Ranger 6.0.0 (10x Genomics). Subsequently, the four samples were combined using the aggr command. Most of the quality control, normalization, dimensionality reduction and clustering were performed based on utilities provided in the Seurat package, version 4.1.0 [8]. Analyses performed on the R platform were performed on R version 4.2.1 [9]. The raw data were filtered to include cells that had at least 200 genes detected and at least 500 unique molecular identifiers (UMIs). Furthermore, only protein-coding genes detected in at least 10 cells were used for further analysis. The biotype of each gene was obtained by querying the Ensembl database and hsapiens_gene_ensembldataset with the help of the useMart and getBM functions provided by the biomaRt package version 2.48.3 [10, 11]. Finally, 14,180 genes and 15,825 cells were retained for further analyses. Regularized negative binomial regression was applied with the use of Seurat's SCTransform function [12, 13] to normalize the data. The SCTransform function also performed variable gene selection and data scaling. When the SCTransform method was applied, cell scores for cell cycle genes were normalized to account for differences in cell cycle stages of the cells.

Integration for batch correction based on the sequencing libraries was performed on the SCT-transformed data with the use of reverse principal components analysis (PCA) as the dimensionality reduction method. PCA was performed with the use of Seurat's RunPCA function to reduce the dimensions in the dataset. RunPCA was performed on 100 principal components. The first 42 principal components were used in Seurat's RunUMAP function to calculate the Uniform Manifold Approximation and Projection representation of the dataset [14]. Clustering was performed using the original Louvain algorithm [15] implemented in Seurat's FindClusters function.

Reanalysis of published results from CRC (GSE132465): A similar approach was used to cluster the single cell RNA-seq data set of colorectal cancer and normal colon samples published by Lee et al. with the accession number GSE132465 [1] to extract the fibroblast population from 57,927 single cells that met our quality criteria. To annotate the identity of the clustered *in vitro* cells, a reference-based integration and label transfer method implemented in Seurat was applied [16]. The pre-annotated stromal subclusters of the public dataset were further subclustered to reveal fibroblast subpopulations. The identified fibroblast subpopulations were used as an integrated reference to label the cultured fibroblast clusters. Outstanding marker genes, heatmaps of marker genes and over-representation analyses of cluster marker genes using the R package ReactomePA version 1.26.0 were used to perform the over-representation analysis [6]. To elucidate the marker genes of each fibroblast subpopulation, a Wilcoxon rank sum test was applied on the normalized RNA data, with Bonferroni correction, with the help of Seurat's FindMarkers function. Pathway activity estimation on the fibroblasts was performed using single-sample gene set enrichment analysis (ssGSEA). The getMsigdb function from the msigdb R package version 1.2.0 [17] was used to download molecular signatures database (MSigDB) version 7.4 [18] hosted on the ExperimentHub version 2.2.1 [19]. Expression of NAF and CAF markers identified by bulk RNA-seq in fibroblast subpopulations was assessed by imputation.

Reanalysis of published results from CRC (GSE178341): In the same manner the dataset GSE178341 [2] was processed to confirm the expression of ADH1B and ITGA3 in NAFs and

CAFs. A total of 267489 cells were retained after filtering of which 68630 were from healthy tissue and 198859 from tumor tissue. In order to focus on informative features, we restricted analyses to protein-coding genes retrieved from the Ensembl April-2020 archived site (apr2020.archive.ensembl.org) [20]. Cell-cycle phase scores (S and G2/M) were computed from canonical gene sets (SeuratData/cc.genes.updated.2019) and inspected to ensure no artifactual clustering by phase. Normalization and variance stabilization were performed per batch using SCTransform with and regression of the cell-cycle difference. Random seeds were set for reproducibility. To mitigate batch effects across chemistry versions used for library preparation, we applied Seurat's SCT-based integration workflow. Principal components analysis PCA was run per batch, and reciprocal PCA (RPCA) anchors were computed. The integrated object was scaled as needed and subjected to PCA. Principal components were retained based on variance inspection. Neighbors were computed on the integrated PCA space, followed by graph-based clustering using a Louvain algorithm. Initial broad cell-type labels were adopted from the original study's annotations (Pelka et al., 2021: GSE178341), in which the stromal compartment comprised endothelial cells, fibroblasts, smooth-muscle cells, perivascular cells, and Schwann cells. We independently confirmed these identities by differential expression. Stromal cells were subset from the integrated object and re-processed (SCTransform, PCA, neighbors, UMAP, clustering) within the subset to refine annotations. Fibroblasts were further subclustered into 1208 CAFs and 2149 NAFs.

Fibroblast functional-state signature scoring: To quantify functional properties of fibroblast subpopulations in the dataset of Lee et al., we computed per-cell, rank-based signature scores using UCell (version 2.12.0) [21]. Gene sets were retrieved via msigdbr (version 25.1.0) from MSigDB [8-9] and grouped into: (i) proliferation (Hallmark: E2F Targets, G2M Checkpoint, MYC Targets v1/v2, Mitotic Spindle), (ii) contractility proxies (Reactome: Smooth Muscle Contraction, Integrin Cell Surface Interactions), (iii) immunomodulatory/MHC-II programs (GO:BP: Cytokine-mediated signaling, Regulation of immune system process, Antigen processing & presentation via MHC class II), and (iv) ECM/fibrosis (Reactome ECM modules; NABA Core Matrisome/Collagens/ECM Glycoproteins/Proteoglycans; GO:BP ECM terms) [22-

25]. Prior to scoring, each signature was intersected with the set of expressed genes in the fibroblast object.

Fibroblast pseudotime analysis: We performed trajectory inference on fibroblasts using Monocle3 (version 1.4.26) [26]. The Seurat UMAP coordinates were transferred into a Monocle3 cell_data_set, after which we ran cell clustering and fitted a principal graph to our UMAP using Monocle3's learn_graph with a single partition. Cells were ordered, anchoring the root to PI16-NAFs. Monocle3 pseudotime values were rescaled to [0,1].

CMS association of CAF signatures

We analyzed GSE39582 (Affymetrix GPL570) in R. The series matrix was retrieved with GEOquery(version 2.76.0) [27] and parsed as an ExpressionSet (Biobase version 2.68.0) [28]. When available, only tumor samples were retained. Expression values were inspected and $\log_2$-transformed as needed. Probes were mapped to ENTREZ IDs using AnnotationDbi (version 1.70.0) with hgu133plus2.db (version 3.13.0) [28]. For CMS subtyping, we applied CMSclassifier (version 1.0.0) [29] using both the Random Forest (RF) and SSP approaches. A single CMS label per sample was assigned by preferring the "nearest" RF class (else RF predicted, else SSP), yielding CMS1–CMS4. For gene-set scoring, ENTREZ IDs were converted to HGNC symbols, and single-sample GSEA scores were computed with GSVA (version 3.22) [5] in ssGSEA (single sample gene set enrichment analysis) [18] mode on curated CAF signatures (myCAF, piCAF). Signatures were intersected with available genes before scoring.

To test associations between CAF subtypes and CMS class, we modeled ssGSEA scores by CMS using a Kruskal–Wallis rank-sum test per signature (non-parametric one-way test across CMS1–CMS4) and performed Post-hoc Dunn tests (BH-adjusted p-values) to identify which CMS pairs differ in that signature (e.g., CMS4 vs CMS2).

Western blot

Fibroblasts were grown in T75 flasks until they reached 90% confluency. The cells were then trypsinized, washed and lysed in RIPA buffer (50 mM Tris-HCl, pH 8.0, 150 mM NaCl, 0.1% SDS, 0.5% sodium dodecyl sulfate (SDS), 1% Igepal) supplemented with 1x complete protease inhibitor cocktail (Roche, #4693116001). Western blotting was carried out as previously described, using 10-50 µg of protein depending on the target [30]. Quantification was conducted via ImageQuant TL v8.1 software (Cytiva, Marlborough, Massachusetts, USA), and the signal intensity was normalized to the GAPDH signal. The following primary antibodies were used for the detection of the target proteins: anti-RRM2 (Invitrogen, Carlsbad, California, USA; #PA5-27856, RRID:AB_2545332, 1:100), anti-MCAM (BD Bioscience, Franklin Lakes, New Jersey, USA; #[P1H12] (A85909), RRID:AB_2752978, 1:500), anti-ADH1B (OriGene, Rockville, Maryland, USA; #TA502777, RRID:AB_11126402, 1:500), anti-CYP1B1 (Invitrogen, PA5-28040, RRID:AB_2545516, 1:1000), anti-SORT1 (SCBT, Dallas, Texas, USA; #sc-376561, RRID:AB_11151576, 1:1000), anti-PTGIS (SCBT, #sc-293247, RRID:AB_3095408, 1:250), anti-CFD (abcam, Cambridge, UK; #ab204917, RRID:AB_3095409, 1:1000), anti-ITGA3 (light chain, SCBT, sc-374242, RRID:AB_10985868, 1:50), anti-TNXB (Proteintech, Rosemont, Illinois, USA; #13595-1-AP, RRID:AB_10644124, 1:500), anti-α-SMA (Abcam, #ab5694, RRID:AB_2223021, 1:1000) and anti-GAPDH (Merck, #MAB374, RRID:AB_2107445, 1:40,000).

Immunohistochemistry

Permanent immunohistochemical staining was performed on formalin-fixed paraffin-embedded colorectal cancer and normal colon tissues as described previously [31]. Dewaxed and rehydrated 4 µm sections were subjected to antigen retrieval at pH 9 for CFD and CYP1B1 detection (Dako, Glostrup, Denmark; #S2367), whereas for all other targets, pH 6.1 was used (Dako, #S1699) at 95 °C for 20 min. Endogenous peroxidase was blocked with 7.5% H_2O_2 for 10 min (Sigma–Aldrich, #1.0721.0250), followed by avidin/biotin (Vectorlabs, Newark,

California, USA; #SP-2001) and 2.5% horse serum blocking (Vectorlabs, #30022) for 20 min. The following primary antibodies were added overnight at 4 °C: anti-CXCL14 (Abcam, #ab137541, RRID:AB_3095410, 1:10.000), anti-RRM2 (Invitrogen, #PA5-27856, RRID:AB_2545332 1:2000), anti-AMIGO2 (SCBT, #sc-373699, RRID:AB_10920216, 1:2000), anti-MCAM (abcam, #ab75769, RRID:AB_21433751:500), anti-ADH1B (Origene, #TA502777, RRID:AB_11126402, 1:20.000), anti-CYP1B1 (abcam, #ab185954, RRID:AB_2894869, 1:50.000), anti-SORT1 (abcam, #ab188586, RRID:AB_2755018, 1:400), anti-CFD (SCBT, #sc-376015, RRID:AB_11008481, 1:100), anti-ITGA3 (abcam, #ab131055, RRID:AB_11156484, 1:1000), and anti-TNXB (SCBT, #sc-271594, RRID:AB_10649946, 1:1000). Specificity of the staining was controlled with a respective isotype control staining. The primary antibodies were detected using the Rabbit Vectastain Elite ABC-Kit (Vectorlabs, #PK-7200, RRID:AB_2336828) according to the manufacturer´s instructions. NovaRED (Vectorlabs, #SK-4800, RRID:AB_2336845) was developed for 20 min. Slides were counterstained with hematoxylin (Merck, #1.05174.0500) for 1.5 min and then mounted with mounting medium (Vectorlabs, #H-5000). Pictures were taken using an Aperio VERSA 8 Slide Scanner (Leica Biosystems, Nussloch, Germany).

Fluorescence immunocytochemistry and histochemistry

Fluorescence immunocytochemistry and histochemistry were performed according to previously published procedures [31, 32]. In brief, for fluorescence immunocytochemical staining, 30,000 NAFs or CAFs were seeded in 4-well chamber slides (Corning, #354104) and permitted to adhere for at least 72 h. Then, the cells were washed and fixed with 4% paraformaldehyde (Sigma–Aldrich, #HT501128) for 15 min. Permeabilization was achieved by incubation with 0.1% Triton-X 100 (Fluka, Buchs, Switzerland; #93426) for 30 min. This was followed by 10 min of blocking with 10% goat serum before incubation with the primary antibodies. For immunohistochemical staining of the tissue slides, the Triton-X 100 concentration was increased to 0.5%, and additional blocking with 3% $H_2 0_2$ (Supelco,

Bellefonte, Pennsylvania, USA; #107210) was conducted for 60 min prior to 1 h of serum blocking. The following primary antibodies were used: anti-α-SMA (abcam, #ab5694, RRID:AB_2223021, 1:4000), anti-Vimentin (abcam, #ab92547, RRID: AB_10562134, 1:10.000), anti-TGM2 (Invitrogen, #MA5-12739, RRID:AB_10985077, 1:1000), anti-ADH1B (OriGene, #TA502777, RRID:AB_11126402, 1:4000), anti-MMP1 (Millipore, Burlington Massachusetts; #IM35, RRID: AB_2282006), anti-ADAMDEC1 (Proteintech, #17899-1-AP, RRID: AB_2878461, 1:500) and anti-ITGA3 (abcam, #ab11767, RRID:AB_2129770, 1:500). The specificity of the staining was controlled via the following isotype antibodies: anti-rabbit IgG (Sigma–Aldrich, #AB-105-c, RRID:AB_354266), anti-mouse IgG2a (R&D systems, #MAB003, RRID:AB_357345) and anti-mouse IgG1 (R&D systems, #MAB002, RRID: AB_357344). Signal detection was conducted via anti-mouse and anti-rabbit ALEXA Fluor 488 Tyramide SuperBoost Kits (Thermo Fisher, #B40912, RRID:AB_2936517 and #B40922, RRID:AB_2941012) and ALEXA Fluor 546 Tyramid conjugates (Thermo Fisher, #B40954) following the manufacturer´s instructions and developing each target for 10 min. Subsequently, the reaction was terminated for 3 min. Nuclei were visualized using DAPI at 1:5000 (Invitrogen, #D21490) or DRAQ5 at 1:800 (Cell Signaling Technology, Danvers, Massachusetts, USA; #4083), and the slides were covered with fluorescence mounting medium (Dako, #S3023).

Survival and correlation analyses

Correlations among CAF signature genes were analyzed using the GEPIA2 portal (Gene Expression Profiling Interactive Analysis) (http://gepia2.cancer-pku.cn/) based on the COAD and READ tumor cohorts, applying Spearman's rank correlation coefficients. Survival analyses were performed using a combined cohort of 16 CRC datasets (n = 1336 for RFS; n = 1061 for OS) encompassing all tumor stages [33]. For each gene or signature, patients were dichotomized into high and low expression groups using a pre-specified cutoff which was applied consistently across all corresponding analyses (all stages, stages I–II, and stages III–IV), as specified in the respective figure legends. Cases with unknown tumor stage were

included in the total cohort analyses but excluded from stage-specific analyses. Consequently, the combined number of patients analyzed in the non-metastatic (UICC I–II) and metastatic (UICC III–IV) subgroups is slightly lower than that of the total cohort. Differences in survival were evaluated by the log-rank test, with $P < 0.05$ considered statistically significant.

References supplementary information

1. Lee HO, Hong Y, Etlioglu HE, Cho YB, Pomella V, Van den Bosch B *et al.* Lineage-dependent gene expression programs influence the immune landscape of colorectal cancer. *Nat Genet*. **52,** 594-603 (2020)

2. Pelka K, Hofree M, Chen JH, Sarkizova S, Pirl JD, Jorgji V *et al.* Spatially organized multicellular immune hubs in human colorectal cancer. *Cell*. **184,** 4734-52 e20 (2021)

3. Stehr AM, Wang G, Demmler R, Stemmler MP, Krug J, Tripal P *et al.* Neutrophil extracellular traps drive epithelial-mesenchymal transition of human colon cancer. *J Pathol*. **256,** 455-67 (2022)

4. Schuhwerk H, Menche C, Armstark I, Gupta P, Fuchs K, Roey Rv *et al.* ZEB1-dependent modulation of fibroblast polarization governs inflammation and immune checkpoint blockade sensitivity in colorectal cancer. *bioRxiv*. 2023.03.28.534565 (2023)

5. Hanzelmann S, Castelo R, Guinney J GSVA: gene set variation analysis for microarray and RNA-seq data. *BMC Bioinformatics*. **14,** 7 (2013)

6. Yu G, He QY ReactomePA: an R/Bioconductor package for reactome pathway analysis and visualization. *Mol Biosyst*. **12,** 477-9 (2016)

7. Jew B, Alvarez M, Rahmani E, Miao Z, Ko A, Garske KM *et al.* Accurate estimation of cell composition in bulk expression through robust integration of single-cell information. *Nat Commun*. **11,** 1971 (2020)

8. Hao Y, Hao S, Andersen-Nissen E, Mauck WM, 3rd, Zheng S, Butler A *et al.* Integrated analysis of multimodal single-cell data. *Cell*. **184,** 3573-87 e29 (2021)

9. Team RC R: A language and environment for statistical computing. (2022)

10. Durinck S, Spellman PT, Birney E, Huber W Mapping identifiers for the integration of genomic datasets with the R/Bioconductor package biomaRt. *Nat Protoc*. **4,** 1184-91 (2009)

11. Durinck S, Moreau Y, Kasprzyk A, Davis S, De Moor B, Brazma A *et al.* BioMart and Bioconductor: a powerful link between biological databases and microarray data analysis. *Bioinformatics*. **21,** 3439-40 (2005)

12. Hafemeister C, Satija R Normalization and variance stabilization of single-cell RNA-seq data using regularized negative binomial regression. *Genome Biol*. **20,** 296 (2019)

13. Choudhary S, Satija R Comparison and evaluation of statistical error models for scRNA-seq. *Genome Biol*. **23,** 27 (2022)

14. McInnes L, Healy J UMAP: Uniform Manifold Approximation and Projection for Dimension Reduction. (2018)

15. Traag VA, Waltman L, van Eck NJ From Louvain to Leiden: guaranteeing well-connected communities. *Sci Rep*. **9,** 5233 (2019)

16. Stuart T, Butler A, Hoffman P, Hafemeister C, Papalexi E, Mauck WM, 3rd *et al.* Comprehensive Integration of Single-Cell Data. *Cell*. **177,** 1888-902 e21 (2019)

17. Bhuva D, Smyth G, Garnham A msigdb: An ExperimentHub Package for the Molecular Signatures Database (MSigDB). (2021)

18. Subramanian A, Tamayo P, Mootha VK, Mukherjee S, Ebert BL, Gillette MA *et al.* Gene set enrichment analysis: a knowledge-based approach for interpreting genome-wide expression profiles. *Proc Natl Acad Sci U S A*. **102,** 15545-50 (2005)

19. Morgan M, Shepherd L ExperimentHub: Client to access ExperimentHub resources. (2022)

20. Yates AD, Achuthan P, Akanni W, Allen J, Allen J, Alvarez-Jarreta J *et al.* Ensembl 2020. *Nucleic Acids Res*. **48,** D682-D8 (2020)

21. Andreatta M, Carmona SJ UCell: Robust and scalable single-cell gene signature scoring. *Comput Struct Biotechnol J*. **19,** 3796-8 (2021)

22. Milacic M, Beavers D, Conley P, Gong C, Gillespie M, Griss J *et al.* The Reactome Pathway Knowledgebase 2024. *Nucleic Acids Res*. **52,** D672-D8 (2024)

23. Ashburner M, Ball CA, Blake JA, Botstein D, Butler H, Cherry JM *et al.* Gene ontology: tool for the unification of biology. The Gene Ontology Consortium. *Nat Genet*. **25,** 25-9 (2000)

24. Gene Ontology C, Aleksander SA, Balhoff J, Carbon S, Cherry JM, Drabkin HJ *et al.* The Gene Ontology knowledgebase in 2023. *Genetics*. **224,** (2023)

25. Naba A, Clauser KR, Hoersch S, Liu H, Carr SA, Hynes RO The matrisome: in silico definition and in vivo characterization by proteomics of normal and tumor extracellular matrices. *Mol Cell Proteomics*. **11,** M111 014647 (2012)

26. Trapnell C, Cacchiarelli D, Grimsby J, Pokharel P, Li S, Morse M *et al.* The dynamics and regulators of cell fate decisions are revealed by pseudotemporal ordering of single cells. *Nat Biotechnol*. **32,** 381-6 (2014)

27. Davis S, Meltzer PS GEOquery: a bridge between the Gene Expression Omnibus (GEO) and BioConductor. *Bioinformatics*. **23,** 1846-7 (2007)

28. Huber W, Carey VJ, Gentleman R, Anders S, Carlson M, Carvalho BS *et al.* Orchestrating high-throughput genomic analysis with Bioconductor. *Nat Methods*. **12,** 115-21 (2015)

29. de Back TR, Wu T, Schafrat PJ, Ten Hoorn S, Tan M, He L *et al.* A consensus molecular subtypes classification strategy for clinical colorectal cancer tissues. *Life Sci Alliance*. **7,** (2024)

30. Krug J, Rodrian G, Petter K, Yang H, Khoziainova S, Guo W *et al.* N-glycosylation Regulates Intrinsic IFN-gamma Resistance in Colorectal Cancer: Implications for Immunotherapy. *Gastroenterology*. **164,** 392-406 e5 (2023)

31. Naschberger E, Liebl A, Schellerer VS, Schutz M, Britzen-Laurent N, Kolbel P *et al.* Matricellular protein SPARCL1 regulates tumor microenvironment-dependent endothelial cell heterogeneity in colorectal carcinoma. *J Clin Invest*. **126,** 4187-204 (2016)

32. Langer V, Vivi E, Regensburger D, Winkler TH, Waldner MJ, Rath T *et al.* IFN-γ drives inflammatory bowel disease pathogenesis through VE-cadherin–directed vascular barrier disruption. *Journal of Clinical Investigation*. **129,** 4691-707 (2019)

33. Gyorffy B Integrated analysis of public datasets for the discovery and validation of survival-associated genes in solid tumors. *Innovation (Camb)*. **5,** 100625 (2024)
